# Segregation of endosymbionts in complex symbiotic system of cicadas providing novel insights into microbial symbioses and evolutionary dynamics of symbiotic organs in sap-feeding insects

**DOI:** 10.1186/s12983-024-00536-0

**Published:** 2024-06-11

**Authors:** Zhi Huang, Dandan Wang, Jinrui Zhou, Hong He, Cong Wei

**Affiliations:** 1 Key Laboratory of Plant Protection Resources and Pest Management of the Ministry of Education, Key Laboratory of Integrated Pest Management On Crops in Northwest Loess Plateau of Ministry of Agriculture and Rural Affairs, College of Plant Protection, Northwest A&F University, Yangling, Shaanxi 712100 China; 2 Key Laboratory of National Forestry and Grassland Administration for Control of Forest Biological Disasters in Western China, College of Forestry, Northwest A&F University, Yangling, Shaanxi 712100 China

**Keywords:** *Hodgkinia*, *Sulcia*, YLS, Bacteriomes, Fat bodies, Sap-feeding insects

## Abstract

The most extraordinary systems of symbiosis in insects are found in the suborder Auchenorrhyncha of Hemiptera, which provide unique perspectives for uncovering complicated insect-microbe symbiosis. We investigated symbionts associated with bacteriomes and fat bodies in six cicada species, and compared transmitted cell number ratio of related symbionts in ovaries among species. We reveal that *Sulcia* and *Hodgkinia* or a yeast-like fungal symbiont (YLS) are segregated from other host tissues by the bacteriomes in the nymphal stage, then some of them may migrate to other organs (i.e., fat bodies and ovaries) during host development. Particularly, YLS resides together with *Sulcia* in the “symbiont ball” of each egg and the bacteriomes of young-instar nymphs, but finally migrates to the fat bodies of adults in the majority of *Hodgkinia*-free cicadas, whereas it resides in both bacteriome sheath and fat bodies of adults in a few other species. The transmitted *Sulcia*/YLS or *Sulcia*/*Hodgkinia* cell number ratio in ovaries varies significantly among species, which could be related to the distribution and/or lineage splitting of symbiont(s). *Rickettsia* localizes to the nuclei of bacteriomes and fat bodies in some species, but it was not observed to be transmitted to the ovaries, indicating that this symbiont may be acquired from environments or from father to offspring. The considerable difference in the transovarial transmission process of symbionts suggests that cellular mechanisms underlying the symbiont transmission are complex. Our results may provide novel insights into insect-microbe symbiosis.

**Supplementary Information:**

The online version contains supplementary material available at 10.1186/s12983-024-00536-0.

## Introduction

Mutually beneficial associations between insects and their symbionts are especially widespread in nature [1–7]. This is particularly the case in insects of the order Hemiptera, which exclusively feed on nutritionally-deficient plant sap [8–10]. Sap-feeding hemipterans usually establish an intimate symbiosis with heritable symbionts that supplement their nutritionally unbalanced diet with essential nutrients [11, 12]. The most extraordinary systems of symbiosis in insects are found in the suborder Auchenorrhyncha of Hemiptera, including spittlebugs, leafhoppers, treehoppers, planthoppers and cicadas [12].

It is generally thought that the auchenorrhynchan ancestor developed an intimate symbiosis with a *Bacteroidetes* currently known as “*Candidatus* Sulcia muelleri” (hereafter referred to as *Sulcia*) and a coresident betaproteobacterium [12–14]. *Sulcia* is harbored in the majority of auchenorrhynchan insects, of which the genome is highly conserved [15]. The ancient betaproteobacterium was still retained in some sap-sucking groups, whereas it has been replaced by other bacteria or fungi in other groups [16–20]. The coresident proteobacterium varied in different sap-feeding groups, e.g., “*Candidatus* Vidania fulgoroideae” (hereafter referred to as *Vidania*) in some planthoppers [21, 22], and “*Candidatus* Hodgkinia cicadicola” (hereafter referred to as *Hodgkinia*) in some cicadas [23–26]. Most auchenorrhynchans have developed highly specialized cells and organs for harboring microbial symbionts, termed bacteriocytes and bacteriomes [13, 14, 16, 17].

In the cicada species that are associated with both *Sulcia* and *Hodgkinia*, *Sulcia* and *Hodgkinia* cells are harbored in the peripheral bacteriocytes and central bacteriocytes, respectively [20]. *Sulcia*’s genome encodes biosynthetic pathways for producing eight essential amino acids, whereas *Hodgkinia*’s genome is complementarily retained for the synthesis of the remaining two essential amino acids and vitamins for the host cicadas [15]. In some cicada species of the genera *Magicicada* and *Tettigades*, *Hodgkinia* has split into two or more complex cytologically distinct but metabolically interdependent cellular lineages [27, 28]. The increase in *Hodgkinia* lineages may present intergenerational transmission problems for host cicadas [29]. A previous study suggests that an important host adaptation to the increase in *Hodgkinia* lineages is an increase in the number of *Hodgkinia* cells transmitted to each egg during the vertical transmission of these symbionts [29].

However, some cicada species lack *Hodgkinia* and instead harbor a yeast-like fungal symbiont (YLS) [20, 30]. Genome sequencing of the fungal symbiont harbored in *Meimuna opalifera* indicates that the YLS genome encodes biosynthetic pathways for synthesizing almost all essential amino acids and other nutrients, which is sufficient to compensate for the lack of *Hodgkinia* [20]. YLS cells are only harbored in the fat bodies among the majority of *Hodgkinia*-free cicada species (e.g., *Cryptotympana atrata*), whereas they are harbored in both the fat bodies and bacteriome sheath in a few *Hodgkinia*-free cicada species, such as *Hyalessa maculaticollis* and *Graptopsaltria tienta* [20, 30, 31]. According to the distribution of *Sulcia*, *Hodgkinia* and YLS, we provide a schematic diagram (Fig. S1) to summarize the distribution of these obligate symbionts in the bacteriomes and fat bodies of cicadas. An early study described that YLS is harbored in the fat bodies of leafhoppers *Ledra auditura* and *Tituria angulata*, whereas *Sulcia* and the coresident bacterial symbiont have been completely lost, and the bacteriomes are even absent in these two species [32]. These observations suggest intimate associations between the microbial symbionts and the evolution of symbiotic organ(s) in sap-feeding insects. However, few studies have been conducted on the evolutionary dynamics of bacteriomes and fat bodies in such insects.

Host insects can acquire symbionts (e.g., several facultative symbionts) from the environment each generation, and they also evolve complex mechanisms to ensure reliable transmission of certain symbionts (e.g., obligate symbionts and a few facultative symbionts) between generations [33–38]. The host-symbiont associations can become more complicated when one of the ancient symbionts has been replaced by another symbiont, or when the host insects are colonized by two obligate symbionts and additional symbiont(s) [22]. Previous studies based on diagnostic PCR amplification or Illumina genome sequencing briefly reported that some cicada species may be associated with facultative symbionts such as *Rickettsia*, *Spiroplasma*, *Sodalis*, *Arsenophonus* and *Wolbachia*, in addition to the obligate symbionts [20, 30, 39]. Fluorescence microscopy, sequencing-based method and ultrastructural observations clarified that *Arsenophonus* harbored in the bacteriomes of cicada *Eopycna repanda* can be transovarially transmitted together with *Sulcia* and *Hodgkinia* from mother to offspring [25]. A previous study showed that *Arsenophonus* in the leafhopper *Macrosteles laevis* can be transovarially transmitted with *Sulcia* and *Nasuia* between generations, but it is harbored in the cytoplasm of *Sulcia* [18]. Another example is that *Rickettsia* harbored in the fat bodies of cicada *Platypleura kaempferi* can be transovarially transmitted with *Hodgkinia* and *Sulcia* between generations [30].

In regard to *Hodgkinia*-free cicadas, YLS cells are only harbored in the fat bodies among the majority of *Hodgkinia*-free cicada species, whereas they are harbored in both the bacteriome sheath and fat bodies of a few *Hodgkinia*-free cicada species [20, 30, 31]. YLS cells in the fat bodies are released to the hemolymph due to the disintegration of fat bodies [30], but it remains unclear how YLS cells in the bacteriomes are released to the hemolymph. To date, *Hodgkinia* complexity driving host adaptations by changing the transmitted *Sulcia/Hodgkinia* cell number ratio has only been investigated in a few species of *Magicicada* and *Tettigades*, but nothing is known about the transmitted *Sulcia/*YLS cell number ratio in the *Hodgkinia*-free cicadas.

Previous studies on the distribution and transovarial transmission of symbionts were mainly focused on a few widely-distributed cicada species. Little is known about the distribution modes and transmission of symbionts during different developmental stages of the host insects as well as the evolutionary process of symbiotic organs. Here, we investigated the composition and distribution of symbionts in the bacteriomes and fat bodies of adults of six representative cicada species using microscopy and sequencing-based methods, including *Karenia caelatata*, *Tanna* sp., *H. maculaticollis*, *G. tienta*, *Tettigetta* sp. and *Eopycna coelestia*. We explored the distribution patterns and transovarial transmission process of *Hodgkinia*, *Sulcia*, YLS and facultative symbiont(s) in these cicadas, and investigated the transmitted *Sulcia/*YLS and *Sulcia/Hodgkinia* cell number ratio during the transmission process. We further clarified the distribution of *Hodgkinia*, *Sulcia* and YLS in freshly laid eggs, first-instar nymphs and adults of three representative cicada species, *E. repanda*, *H. maculaticollis* and *C. atrata,* which is important for uncovering the intimate associations between the microbial symbionts and the evolutionary dynamics of symbiotic organs. The results may provide new insights into the diversification and transovarial transmission of symbionts and evolutionary processes of symbiotic organs in cicadas and other auchenorrhynchan insects.

## Results

### General features of bacteriomes and fat bodies in the adults of six representative cicada species

Each cicada had two pairs of bacteriomes, residing laterally at the bottom of the coelom of the fifth and sixth abdominal segments, respectively. The bacteriomes were composed of sphere-like clusters termed bacteriome units, which were connected by fine tracheae (Fig. S2). The bacteriome units were surrounded by numerous fat bodies in these cicadas. The shape of each bacteriome unit was morphologically similar within a cicada species, but it exhibited obvious differences among species (Fig. S2). The numbers of bacteriome units were approximate 99, 12, 276, 116, 367 and 235 in *E*. *coelestia*, *Te.* sp., *K*. *caelatata*, *Ta.* sp., *G. tienta* and *H*. *maculaticollis*, respectively (Table S1). However, the numbers of fat bodies were difficult to count due to the unobvious shape of them.

The bacteriome sheath was composed of a single-layered epithelial cells, which varied in wall thickness among different cicadas (Fig. S2, Fig. S3 and Table S1). The bacteriome sheath of *G. tienta* and *H*. *maculaticollis* was thicker than that of* E*. *coelestia*, *Te.* sp., *K*. *caelatata* and *Ta.* sp. (Table S1).

### Distribution of symbionts in bacteriomes and fat bodies in the adults of six representative cicada species

*Sulcia* was detected in the bacteriomes of all six cicada species, and the 16S rRNA gene sequences of *Sulcia* shows high similarity (99.40–99.93%) with that of other cicadas (Tables S2 and S3). The phylogenetic analysis of *Sulcia* clearly showed that all *Sulcia* symbionts form a well-supported monophyletic group, with strong support in both the BI and ML trees (Fig. S4). For *E*. *coelestia* and *Te.* sp. that contained both *Sulcia* and *Hodgkinia*, *Sulcia* and *Hodgkinia* occupied the peripheral and central bacteriocytes of bacteriomes, respectively, and neither of them were observed in the bacteriome sheath and fat bodies (Fig. S3A to H). Notably, 16S rRNA gene sequences of *Hodgkinia* are dissimilar within a species (Tables S2 and S3); 16S rRNA gene sequences of *Hodgkinia* of *E*. *coelestia* and *Te*. sp. exhibit relatively low similarity (96.50–99.93%) to that of other cicadas, but all the *Hodgkinia* symbionts form a well-defined monophyletic group in the phylogenetic trees (Fig. S5). *Sulcia* was harbored in the bacteriomes of *K*. *caelatata* and *Ta.* sp., while YLS only occupied the fat bodies of these two species (Fig. S3I to P). In contrast, *Sulcia* was harbored in the bacteriomes of *G. tienta* and* H*. *maculaticollis*, and YLS was harbored in the bacteriome sheath besides the fat bodies in these two species (Fig. S3Q to X). The 18S rRNA gene sequence of YLS in the bacteriomes was the same as that in the fat bodies for both *G. tienta* and* H*. *maculaticollis* (Tables S2 and S3). The 18S rRNA gene sequences of YLS of four *Hodgkinia*-free cicadas (i.e., *K*. *caelatata*, *Ta.* sp., *G. tienta* and *H*. *maculaticollis*) exhibit high similarity (99.67–100%) with that of other *Hodgkinia*-free cicadas (Table S3). Phylogenetic trees of cicada-parasitizing fungi and cicada fungal symbionts clearly show that some cicada-parasitizing fungi (e.g., *Ophiocordyceps longissima* and *Ophiocordyceps yakusimensis*) are intermingled together with YLS symbionts, whereas the majority of cicada-parasitizing fungi (e.g., O*phiocordyceps sobolifera*) are placed outside the clade of YLS symbionts (Fig. S6).

**Fig. 1:**
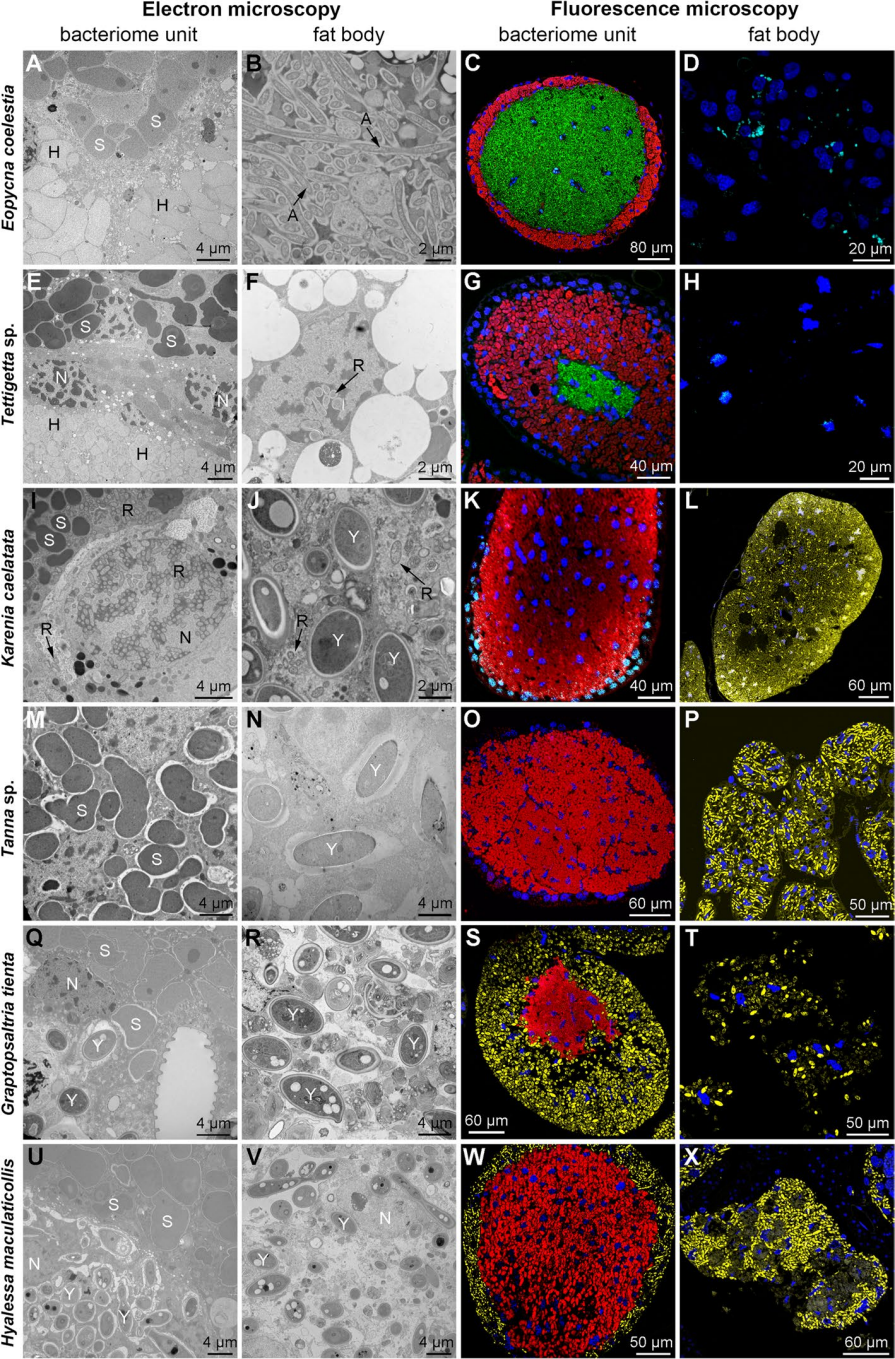
Distribution of *Sulcia*, *Hodgkinia*, YLS and additional symbiont(s) in the bacteriomes and fat bodies of representative cicadas. **A** to **D** For *E. coelestia*, *Sulcia* and *Hodgkinia* in the bacteriomes, and *Arsenophonus* in the cytoplasma of fat bodies. **E** to **H** For *Te.* sp., *Sulcia* and *Hodgkinia* in the bacteriomes, and *Rickettsia* in the nucleus of fat bodies. **I** to **L** For *K. caelatata*, *Sulcia* in the bacteriomes and YLS in the fat bodies, and *Rickettsia* in the bacteriome sheath, bacteriocyte nucleus, and cytoplasma of bacteriocytes and fat bodies. **M** to **P** For *Ta.* sp, *Sulcia* in the bacteriomes and YLS in the fat bodies. **Q** to **T** For *G. tienta*, *Sulcia* in the bacteriomes, and YLS in the fat bodies and bacteriome sheath of bacteriomes. **U** to **X** For *H. maculaticollis*, *Sulcia* in bacteriomes, and YLS in the fat bodies and bacteriome sheath of bacteriomes. For fluorescence microscopy, blue, cyan, yellow, red and green represent nucleus, additional symbiont, YLS, *Sulcia* and *Hodgkinia*, respectively. Abbreviations: BS, bacteriome sheath; A, *Arsenophonus*; H, *Hodgkinia*; N, nucleus; R, *Rickettsia*; S, *Sulcia*; Y, yeast-like fungal symbiont. Black arrows represent *Arsenophonus* or *Rickettsia*

Based on fluorescence and transmission microscopy, we further confirm the distribution of symbionts in bacteriomes and fat bodies of the sampled species. For *E*. *coelestia* and *Te.* sp. that contain *Sulcia* and *Hodgkinia* in the bacteriomes, both *Sulcia* and *Hodgkinia* cells were irregular in shape. Under transmission electron microscopy, *Sulcia* cells were electron-dense, and *Hodgkinia* cells were electron-translucent (Fig. 1A to H). For the four species that contain *Sulcia* and YLS, *Sulcia* cells were irregular in shape, and YLS cells were rod-shaped. In contrast, YLS cells possessed a two-layered cell wall with some cells showing yeast-like budding particles (Fig. 1I to X). *Arsenophonus* was only observed in the cytoplasma of fat bodies in *E*. *coelestia*, but it was not observed in the host nuclei of fat bodies in this species (Fig. 1A to D and Table S4). *Rickettsia* was only harbored in the host nuclei of fat bodies in *Te.* sp., which was not observed in the cytoplasm of fat bodies (Fig. 1E to H and Table S4). In contrast, *Rickettsia* was observed not only in the cytoplasma of fat bodies, the cytoplasma of sheath cells and bacteriocytes of bacteriomes, but also in some host nuclei of bacteriomes in *K*. *caelatata* (Fig. 1I to L and Table S4).

**Fig. 2:**
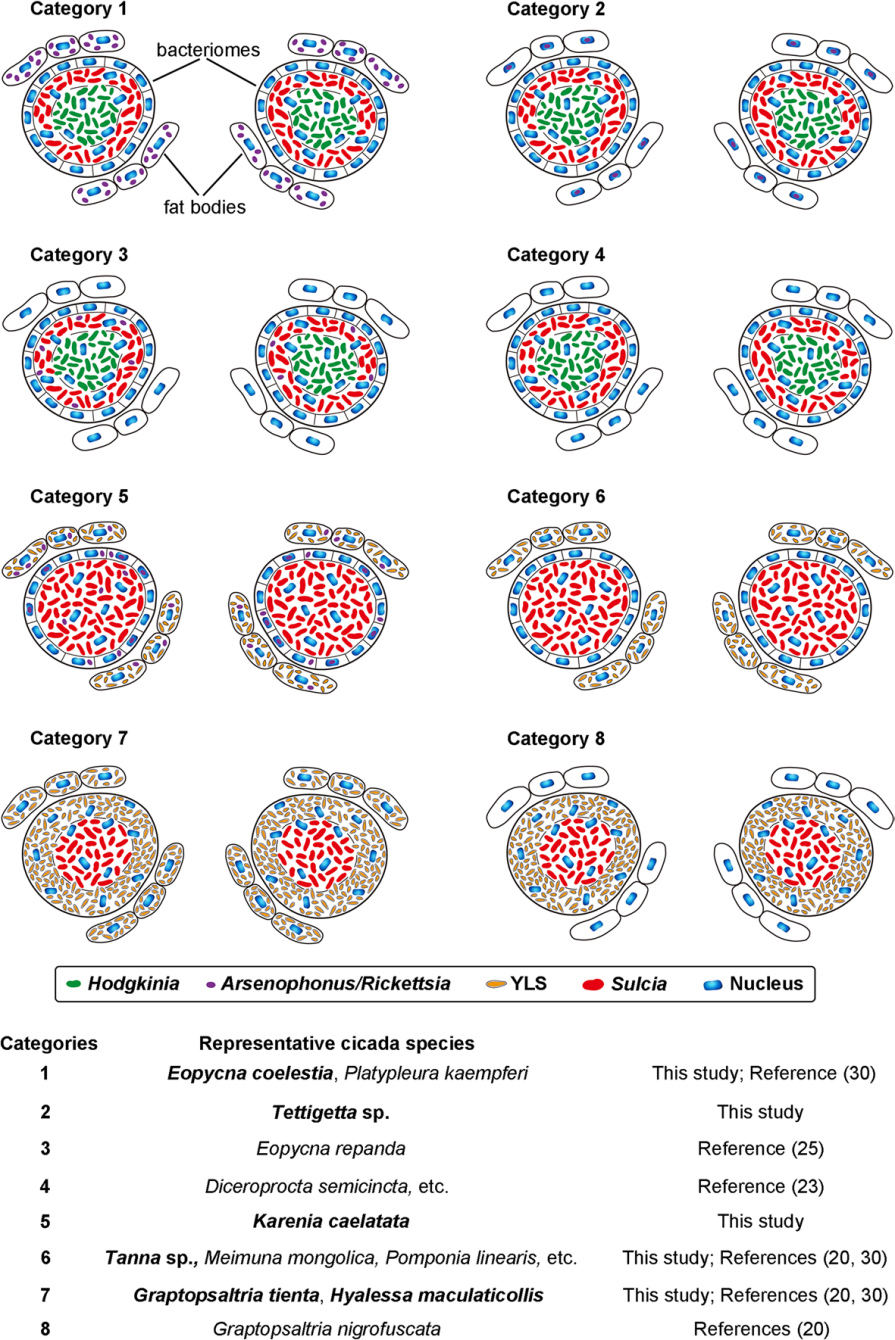
Schematic representation showing the complex distribution of symbionts harbored in cicadas based on these 6 representative cicada species and other 31 cicada species

According to the symbiont diversity and distribution in the bacteriomes and fat bodies of these six representative cicada species, the composition of symbionts can be divided into five categories, which together with other three known categories are schematically illustrated in Fig. 2 and described as follows. Category 1: *Sulcia* and *Hodgkinia* harbored in the bacteriocyte cytoplasma of bacteriomes, and additional symbiont(s) only harbored in the cytoplasma of fat bodies (*E*. *coelestia*); Category 2: *Sulcia* and *Hodgkinia* harbored in the bacteriocyte cytoplasma of bacteriomes, and additional symbiont(s) only harbored in some host nuclei of the fat bodies (*Te.* sp.); Category 3: *Sulcia, Hodgkinia* and additional symbiont(s) are harbored in the bacteriomes, with no symbiont harbored in the fat bodies; Category 4: both *Sulcia* and *Hodgkinia* are harbored in the bacteriomes, with no symbiont harbored in the fat bodies; Category 5: *Sulcia* harbored in the bacteriocyte cytoplasma of bacteriomes, YLS harbored in the cytoplasma of fat bodies, and additional symbiont(s) harbored in the cytoplasma of fat bodies, the cytoplasma of sheath cells and bacteriocyte cytoplasma of bacteriomes, and some host nuclei of bacteriomes (*K*. *caelatata*); Category 6: *Sulcia* harbored in the bacteriocyte cytoplasma of bacteriomes, and YLS harbored in the cytoplasma of fat bodies (*Ta.* sp.); Category 7: *Sulcia* harbored in the bacteriocyte cytoplasma of bacteriomes, and YLS harbored in the well-developed bacteriome sheath cells besides the cytoplasma of fat bodies (*G. tienta* and* H*. *maculaticollis*); Category 8: *Sulcia* and YLS harbored in the bacteriomes, with no symbiont harbored in the fat bodies. In these eight categories, Category 2 and Category 5 are novel for Cicadidae.

### Transovarial transmission of symbionts from mother to offspring in six representative cicada species

**Fig. 3:**
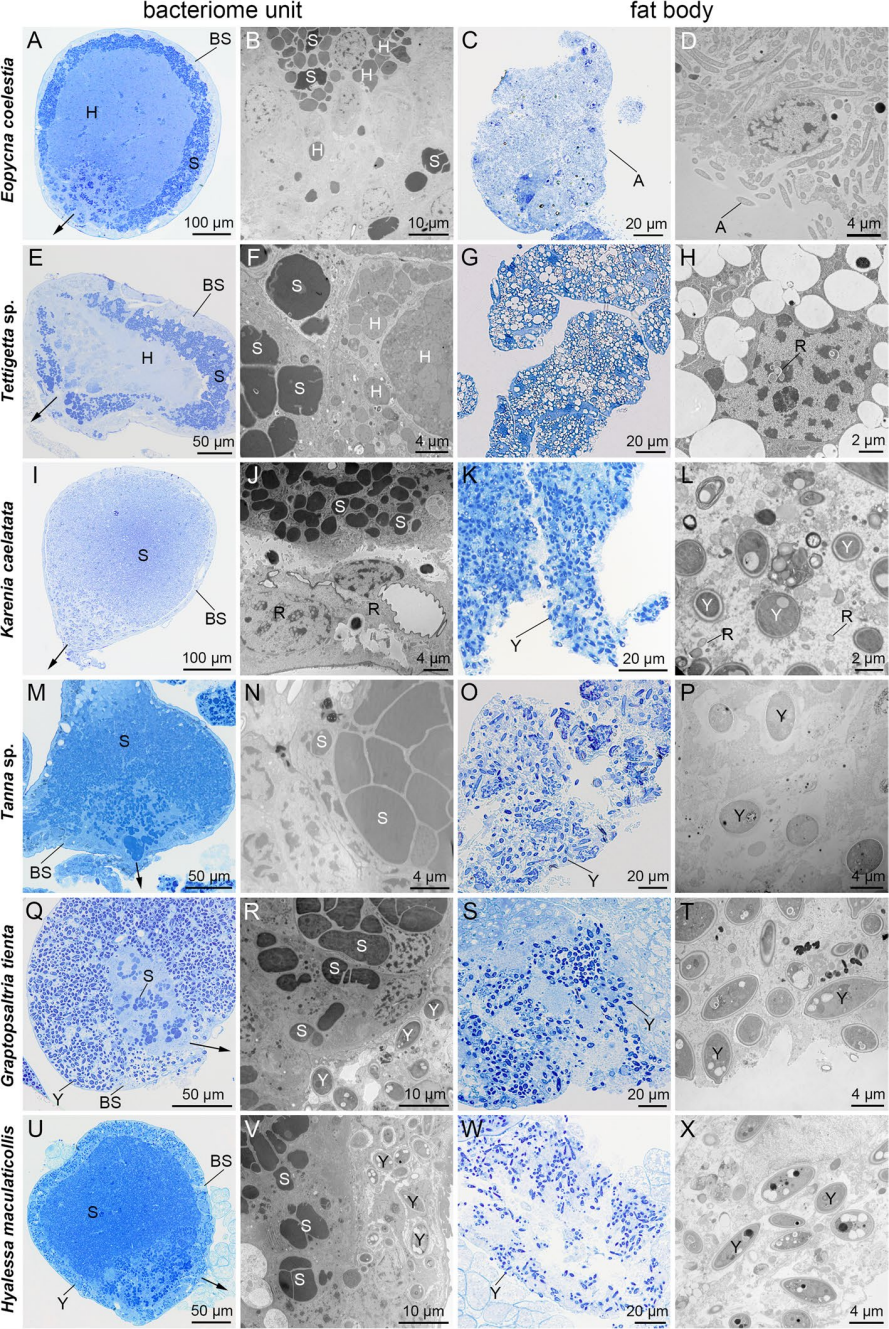
Symbionts in bacteriomes and fat bodies before migration to the hemolymph in different cicada species. **A** to **D** For *E. coelestia*, *Sulcia* and *Hodgkinia* released from the bacteriomes to the hemolymph, and *Arsenophonus* released from fat bodies to the hemolymph. **E** to **H** For *Te.* sp., *Sulcia* and *Hodgkinia* released from bacteriomes to the hemolymph. **I** to **L** For *K. caelatata*, *Sulcia* released from the bacteriomes to the hemolymph, and YLS released from fat bodies to the hemolymph. **M** to **P** For *Ta.* sp., *Sulcia* released from the bacteriomes to the hemolymph, and YLS released from fat bodies to the hemolymph. **Q** to **T** For *G. tienta*, *Sulcia* released from the bacteriomes to the hemolymph, and YLS released from the fat bodies and the bacteriome sheath to the hemolymph. **U** to **X** For *H. maculaticollis*, *Sulcia* released from the bacteriomes to the hemolymph, and YLS released from the fat bodies and the bacteriome sheath to the hemolymph. Abbreviations: BS, bacteriome sheath; A, *Arsenophonus*; H, *Hodgkinia*; N, nucleus; R, *Rickettsia*; S, *Sulcia*; Y, yeast-like fungal symbiont. Black arrows represent the emigration of the symbiont(s)

According to the histological, fluorescence and transmission electron microscopy, we reveal that in *E*. *coelestia* and *Te.* sp., both *Sulcia* and *Hodgkinia* were exocytosed from the bacteriocytes to the intercellular space and finally extruded to the hemolymph (Fig. 3A to H). *Arsenophonus* cells harbored in the cytoplasma of fat bodies of *E*. *coelestia* were released into the hemolymph after the disintegration of fat bodies (Fig. 3C to D). In contrast, *Rickettsia* cells inside the host nuclei of fat bodies of *Te.* sp. were not observed to be released into the hemolymph (Fig. 3G to H). In *K*. *caelatata* and *Ta.* sp., *Sulcia* cells were exocytosed from the bacteriocytes to the intercellular space and finally extruded to the hemolymph, whereas YLS cells were released to the hemolymph after the disintegration of fat bodies (Fig. 3I to P). However, *Rickettsia* cells were not observed being released into the hemolymph from bacteriomes and/or fat bodies in *K*. *caelatata* (Fig. 3I to L). In *G. tienta* and *H*. *maculaticollis*, both *Sulcia* and YLS harbored in the bacteriomes were extruded to the hemolymph, while YLS harbored in the fat bodies were released into the hemolymph after the disintegration of fat bodies (Fig. 3Q to X). *Sulcia* cells or *Sulcia* cells together with *Hodgkinia* cells (when harbored together) in the bacteriomes of cicadas were gathered tightly before being released to the hemolymph, while YLS cells in the bacteriomes of *G. tienta* and *H*. *maculaticollis* did not gather together before being released to the hemolymph (Fig. 3 and Fig. S7).

**Fig. 4:**
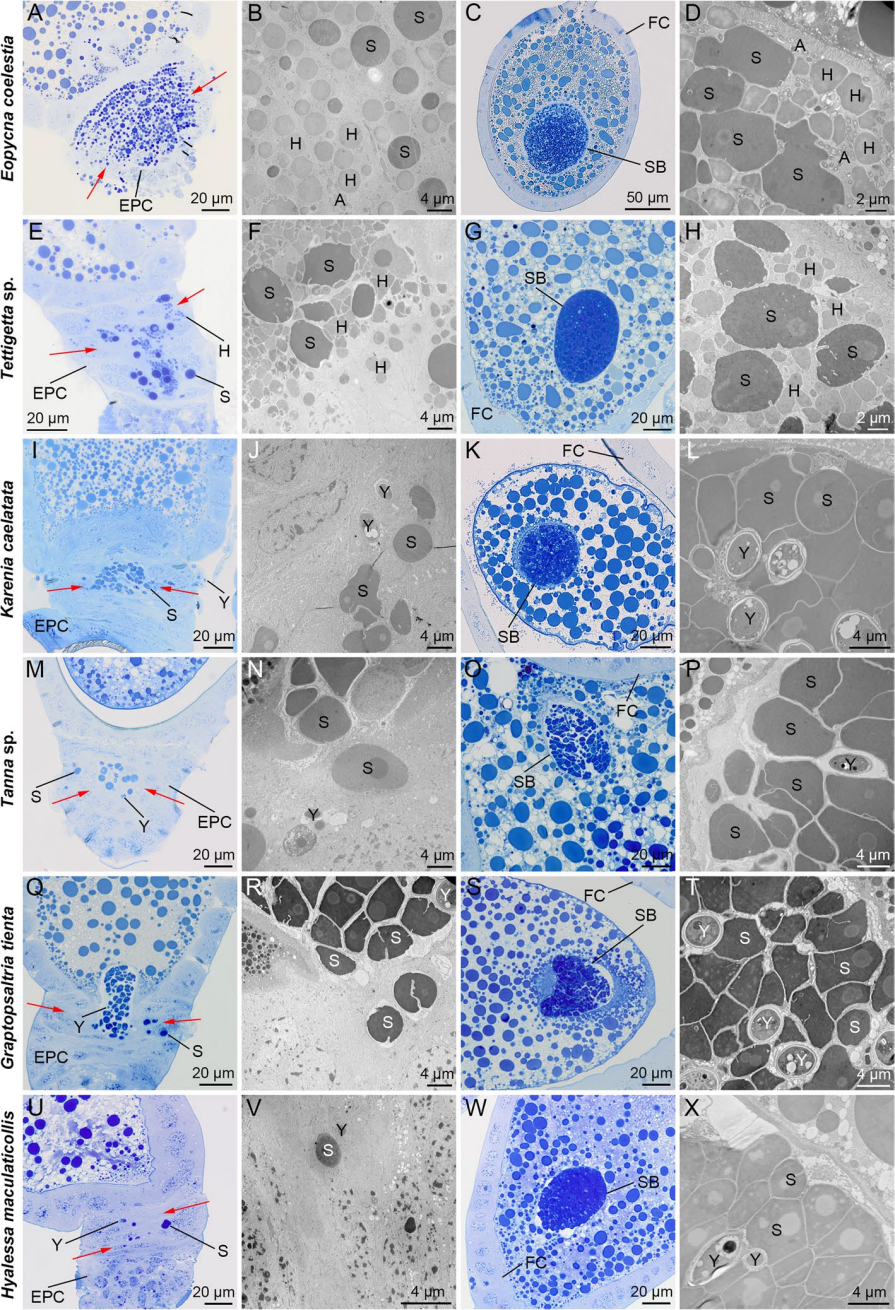
Transmission of symbionts from hemolymph to mature oocytes in different cicada species. **A** to **D** For *E. coelestia*, *Arsenophonus* together with *Sulcia* and *Hodgkinia* were transported to the posterior pole of the mature oocytes, migrating through the cytoplasma of epithelial plug cells into the perivitelline space and finally formed a characteristic “symbiont ball” in each egg. **E** to **H** For *Te.* sp., *Sulcia* and *Hodgkinia* were transported to the posterior pole of the mature oocytes, migrating through the cytoplasma of epithelial plug cells into the perivitelline space and finally formed a “symbiont ball” in each egg. **I** to **L** For *K. caelatata*, *Sulcia* and YLS were transported to the posterior pole of the mature oocytes, migrating through the cytoplasma of epithelial plug cells into the perivitelline space and finally formed a “symbiont ball” in each egg. **M** to **P** For *Ta.* sp., *Sulcia* and YLS were transported to the posterior pole of the mature oocytes, migrating through the cytoplasma of epithelial plug cells into the perivitelline space and finally formed a “symbiont ball” in each egg. **Q** to **T** For *G. tienta*, *Sulcia* and YLS were transported to the posterior pole of the mature oocytes, migrating through the cytoplasma of epithelial plug cells into the perivitelline space and finally formed a “symbiont ball” in each egg. **U** to **X** For *H. maculaticollis*, *Sulcia* and YLS were transported to the posterior pole of the mature oocytes, migrating through the cytoplasma of epithelial plug cells into the perivitelline space and finally formed a “symbiont ball” in each egg. Abbreviations: A, *Arsenophonus*; H, *Hodgkinia*; S, *Sulcia*; Y, yeast-like fungal symbiont; EPC, epithelial plug cell; FC, follicular cell; SB, symbiont ball. Red arrows representing the emigration of the symbionts from hemolymph to perivitelline space

**Fig. 5:**
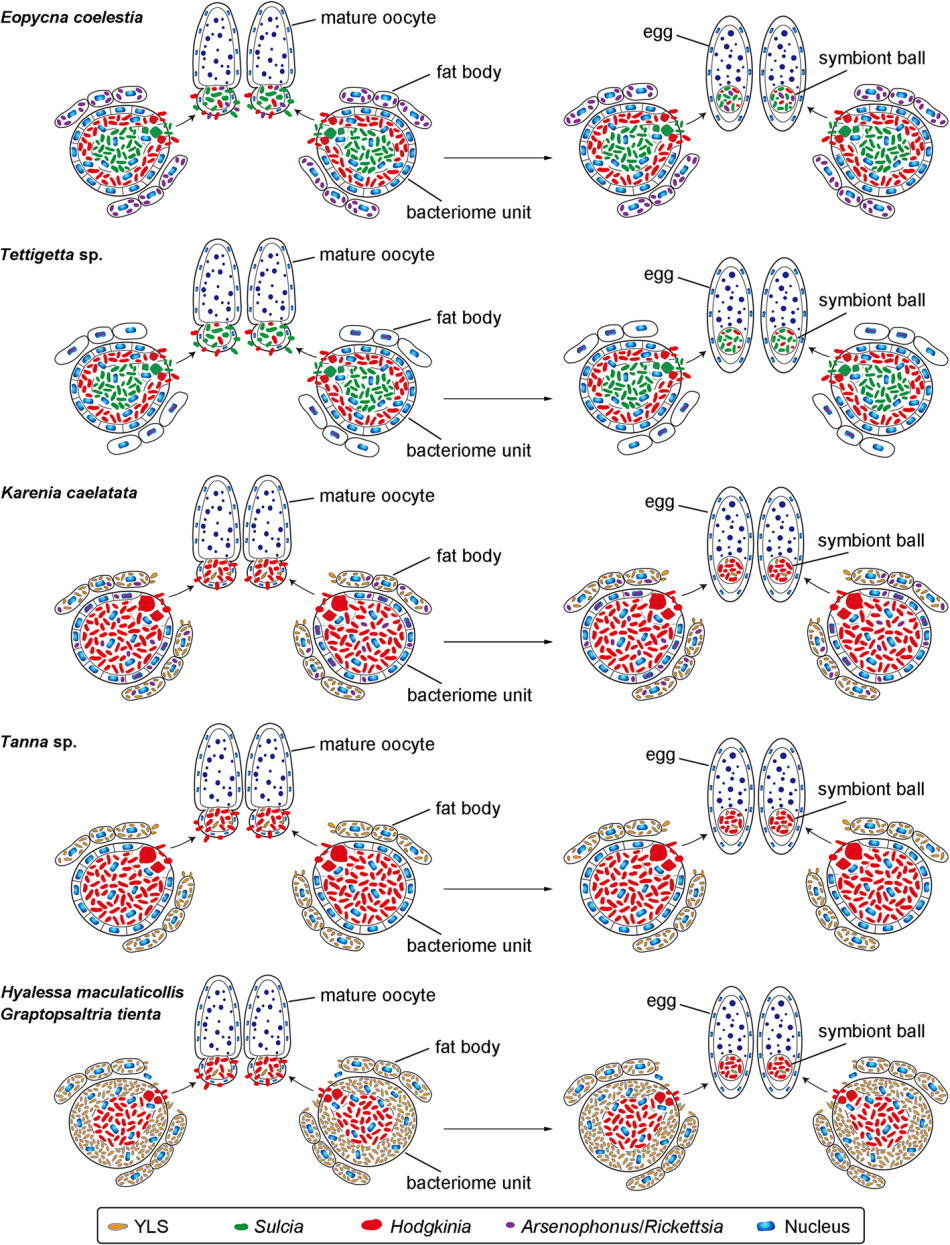
Schematic representation showing the transovarial transmission of symbionts in different cicadas

In *E*. *coelestia* and *Te.* sp., both *Sulcia* and *Hodgkinia* gathered around the posterior pole of terminal oocytes and then migrated through the cytoplasma of epithelial plug cells into the perivitelline space (viz., space between epithelial plug cells and oolemma), and they finally formed a characteristic “symbiont ball” in each egg (Fig. 4A to H and Fig. S8A to H). *Arsenophonus* harbored in the cytoplasma of fat bodies of *E*. *coelestia* were transovarially transmitted with *Sulcia* and *Hodgkinia*. *Rickettsia* harbored in the host nuclei of fat bodies of *Te.* sp. was not observed to be transmitted to the mature oocytes (Fig. 4A to H and Fig. S8A to H). In *K*. *caelatata*, *Ta.* sp., *G. tienta* and* H*. *maculaticollis*, both *Sulcia* and YLS were transported to the posterior pole of the mature oocytes, and then migrated through the cytoplasma of epithelial plug cells into the perivitelline space, and they finally formed a characteristic “symbiont ball” in each egg (Fig. 4I to X and Fig. S8I to X). *Rickettsia* was not observed to be transported to the mature oocytes, although it was found in the bacteriomes and fat bodies of *K*. *caelatata* (Fig. 1I to L and Fig. 4I to L). We provide a schematic representation to illustrate the transovarial transmission process of related symbionts in these six representative cicada species (Fig. 5).

Additionally, statistical analysis was performed to estimate the ratio of the cell numbers of *Sulcia*/*Hodgkinia* and *Sulcia*/YLS transmitted into mature oocytes during the vertical transmission process. We reveal that the ratio of the cell numbers of *Sulcia*/YLS within mature oocytes in each individual was approximate 4.53:1, 4.16:1, 11.90:1 and 23.88:1 in *K*. *caelatata*, *Ta.* sp., *G. tienta* and *H*. *maculaticollis*, respectively (Fig. S9A and Table 1). The ratio of the cell numbers of *Sulcia*/*Hodgkinia* within mature oocytes in each individual was approximate 1:9.50 in *E*. *coelestia* and 1:13.21 in *Te.* sp. (Fig. S9B and Table 1).

**Table 1:** The ratio of transmitted obligate symbionts in mature oocytes of different cicadas

Species	Numbers of cicada individuals observed	Average numbers of symbiont cells per section			Ratio of S:H	Ratio of S:Y
		*Sulcia*	*Hodgkinia*	YLS		
*Karenia caelatata*	8	589	/	130	/	4.53: 1
*Tanna* sp.	8	524	/	126	/	4.16: 1
*Graptopsaltria tienta*	8	571	/	48	/	11.90: 1
*Hyalessa maculaticollis*	8	955	/	40	/	23.88: 1
*Eopycna coelestia*	8	353	3355	/	1: 9.50	/
*Tettigetta* sp.	8	578	7633	/	1: 13.21	/

### Distribution of symbionts in freshly laid eggs, first-instar nymphs and adults of representative cicadas

**Fig. 6:**
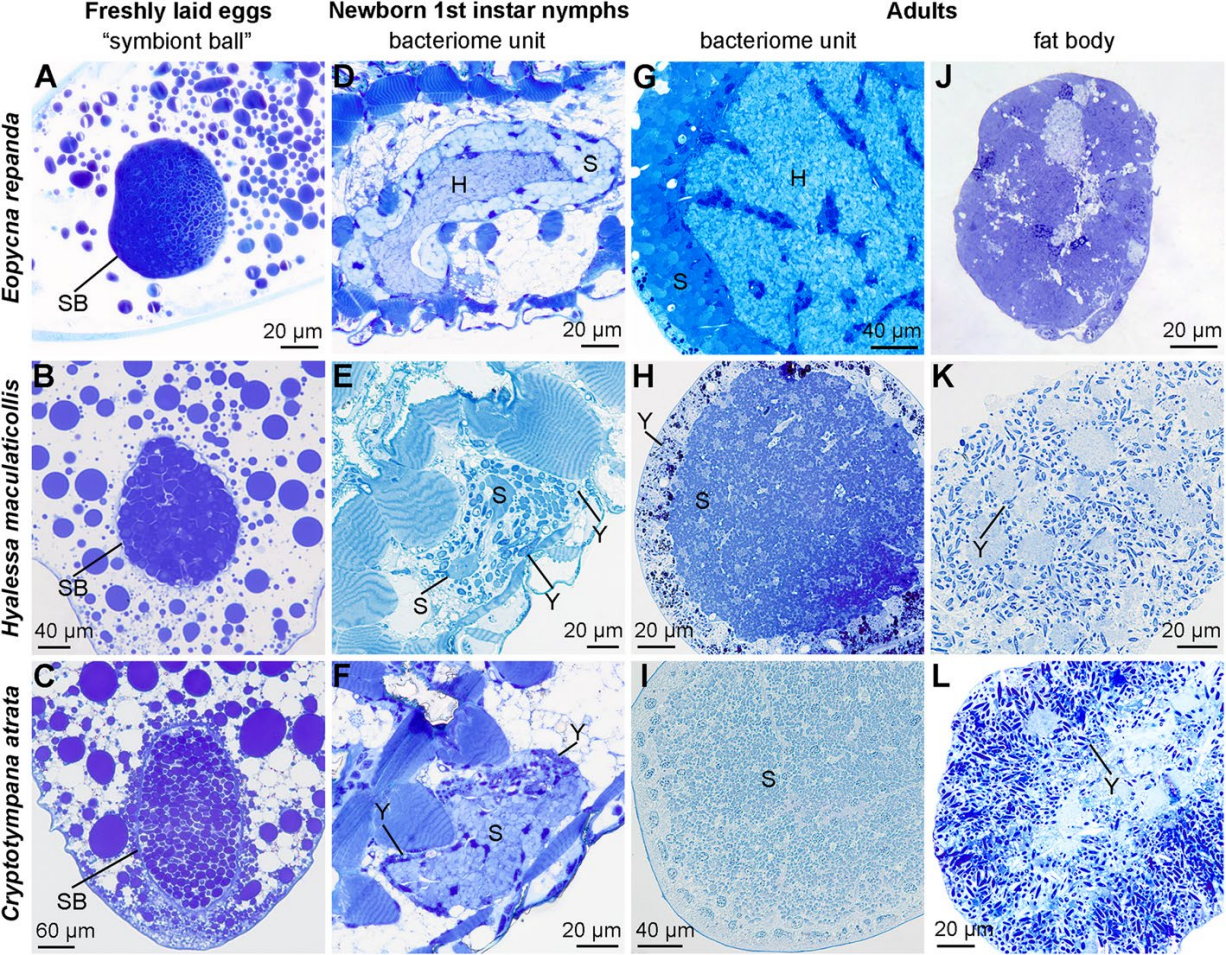
Distribution of *Sulcia*, YLS and *Hodgkinia* in the freshly laid eggs, nymphs and adults of three representative cicada species. **A** to **C** Both *Sulcia* and the coresident symbiont (i.e., YLS or *Hodgkinia*) were intermixed within the “symbiont ball” in the freshly laid eggs of *E. repanda*, *C. atrata* and *H. maculaticollis*. **D** *Sulcia* and *Hodgkinia* were harbored in the bacteriomes of the newborn first-instar nymphs of *E. repanda*. **E** to **F** YLS was initially harbored in the bacteriome sheath of bacteriomes in the newborn first-instar nymphs of *C. atrata* and *H. maculaticollis*. **G** to **L** *Sulcia* was harbored in the bacteriomes of adults of three cicada species, and *Hodgkinia* was present in the bacteriomes of *E. repanda* adults. In regard to the *Hodgkinia*-free cicadas, YLS is harbored in the fat bodies of *C. atrata* adults and in both the bacteriomes and fat bodies of *H. maculaticollis* adults. Abbreviations: H, *Hodgkinia*; S, *Sulcia*; Y, yeast-like fungal symbiont; SB, symbiont ball

Both *Sulcia* and the coresident symbiont (i.e., YLS or *Hodgkinia*) were intermixed within the “symbiont ball” in the freshly laid eggs of *E. repanda*, *C. atrata* and *H. maculaticollis* (Fig. 6A to C). *Sulcia* and *Hodgkinia* were harbored in the bacteriocytes of bacteriomes in the newborn first-instar nymphs of *E. repanda* (Fig. 6D). *Sulcia* was harbored in the bacteriocytes of bacteriomes, whereas YLS was harbored in the bacteriome sheath of bacteriomes in the newborn first-instar nymphs of both *C. atrata* and *H. maculaticollis* (Fig. 6E to F). *Hodgkinia* was present in the bacteriomes of *E. repanda* adults, and *Sulcia* was harbored in the bacteriomes of adults of three cicada species. YLS was harbored in the fat bodies of *C. atrata* adults and in both the bacteriome sheath and fat bodies of *H. maculaticollis* adults (Fig. 6G to L).

## Discussion

### Cicadas’ adaptation to the increase in *Hodgkinia* lineages

Previous studies reported that *Hodgkinia* can fragment into complexes of cytologically distinct lineages with more reduced but complementary genomes in some cicada species of the genera *Magicicada* and *Tettigades* [23, 24]. The increase in *Hodgkinia* lineages may present an intergenerational transmission problem for host cicadas [29]. It has been revealed that the transmitted *Sulcia/Hodgkinia* cell number ratio was nearly 1:1 in cicadas having a single *Hodgkinia* lineage and nearly 1:2.4 in cicadas having six *Hodgkinia* lineages, whereas it was 1:11.2 in the species that harbors the most complex *Hodgkinia* lineages [29]. Our results show that 16S rRNA gene sequences of *Hodgkinia* were dissimilar within a species for *E*. *coelestia* and *Te.* sp. (Table S3). Furthermore, the transmitted *Sulcia*/*Hodgkinia* cell number within mature oocytes was approximate 1: 9.50 and 1: 13.21 in *E*. *coelestia* and *Te.* sp., respectively (Table 1). The reason for these remains unknown. We hypothesize that *Hodgkinia* may have fragmented into cytologically distinct lineages in these two cicadas, which merits further investigation.

### Coexisting of symbionts in cicadas

Previous studies suggest that symbiont evolution can drive compensatory adaption in host insects, such as horizontal gene transfer (HGT) of symbiont genes into the genomes of host insects [39–42]. A systematic investigation of HGT-acquired genes in insect genomes revealed that several insect symbionts (e.g., *Arsenophonus*, *Rickettsia* and *Wolbachia*) are involved in the transitions of related genes into insect genomes of some insect groups [41]. Ultrastructural microscopy revealed that *Rickettsia* was observed to localize to some host nuclei of related host insects, such as the treehopper *Centrotus cornutus* and the leafhopper *Nephotettix cincticeps* [19, 43]. We observed that *Rickettsia* was harbored in the cytoplasma of bacteriome sheath, nuclei and cytoplasma of bacteriocyte of bacteriomes and also cytoplasma of fat bodies in *K*. *caelatata*, and it was only harbored in the nuclei of fat bodies in *Te.* sp. (Fig. 1). Although it has been reported that *Rickettsia* was harbored in the host nuclei of a few insects, the functional role of this symbiont has not yet been validated. Future studies are required to clarify the functions and significance of *Rickettsia* and other facultative symbionts in host nuclei of cicadas and other insects. Additionally, a previous study revealed that *Sulcia* and YLS in the cicada *Subpsaltria yangi* are harbored in the bacteriomes and fat bodies, respectively, but a few OTUs isolated from the bacteriomes were identified as *Hodgkinia* [44]. Whether *Sulcia*, *Hodgkinia* and YLS coexist in *S. yangi* and/or other unexamined cicada species merits further investigation. Hopefully, the novel distribution pattern(s) of symbionts in cicadas would be revealed in the future.

### Host cicadas utilize different strategies to acquire related symbionts

We observed significant differences between the fungal symbiont (i.e., YLS) and bacterial symbionts (i.e., *Sulcia* and *Hodgkinia*) in the transmission process from the bacteriomes to the hemolymph (Fig. S7). In cicada species harboring *Sulcia* and *Hodgkinia* in the bacteriomes, *Sulcia* and *Hodgkinia* cells gather tightly before being released to the hemolymph, whereas YLS harbored in the bacteriomes of *G. tienta* and *H*. *maculaticollis* do not gather together before being released to the hemolymph, and each YLS cell is individually extruded to the hemolymph (Fig. S7). It is noteworthy that YLS cells in the fat bodies are released to the hemolymph due to the disintegration of fat bodies, whereas YLS cells in the bacteriomes are extruded to the hemolymph near the junction of bacteriome units. It has been revealed that the signal pathways of *Sulcia* play important roles in regulating the host/symbiont interaction and symbiont transmission in cicadas and other sap-feeding auchenorrhynchan insects [45]. The cellular and potential molecular mechanisms underlying transmission of YLS from bacteriomes/fat bodies to the hemolymph of insects merits further investigation.

It has been reported that some facultative symbionts can be transovarially transmitted with obligate symbionts by “hitching a ride” [30]. A previous study showed that *Arsenophonus* in the leafhopper *M. laevis* can be transovarially transmitted with *Sulcia* and *Nasuia* between generations, but it is harbored in the cytoplasm of *Sulcia* [18]. In our present study, we revealed that *Arsenophonus* is only harbored in the cytoplasma of fat bodies in *E. coelestia* and can be transovarially transmitted together with *Hodgkinia* and *Sulcia* from mother to offspring (Figs. 3 and 4). It has been revealed that *Sulcia* participates in the biosynthesis of three essential amino acids and *Vidania* involves in synthesizing the remaining seven essential amino acids, and *Arsenophonus* contributes to the biosynthesis of B vitamins in planthopper *Ranissus scytha* [22]. To date, the biological function of *Arsenophonus* in *E. coelestia* and other cicadas (e.g., *E. repanda*) still remain unknown. Future studies are required to clarify whether *Arsenophonus* of *E. coelestia* can participate in the biosynthesis of essential nutrients such as B vitamins. The transovarial transmission and biological functions of facultative symbiont(s) are quite complex in sap-feeding insects. The novel transmission pattern(s) and related functions of facultative symbiont(s) in cicadas need to be investigated in the future.

We revealed that *Rickettsia* is not only harbored in the sheath, cytoplasm and nuclei of the bacteriocytes of bacteriomes, but also in the cytoplasm of fat bodies of *K. caelatata*. In contrast, *Rickettsia* is present only in the nuclei of the fat bodies of *Te.* sp. (Figs. 1 and 2). However, *Rickettsia* was not observed to be transovarially transmitted from mother to offspring in these two cicadas, indicating that this facultative symbiont is possibly acquired from the environment [35, 46, 47]. Notably, it has been reported that *Rickettsia* can hitchhike with insect sperm for intrasperm vertical symbiont transmission in the leafhopper *N. cincticeps* [43]. An alternative acquirement of *Rickettsia* in related cicada species could be that it is transmitted directly from father to offspring. It would be interesting to explore the paternal transmission process and horizontal transmission routes of facultative symbionts (e.g., *Rickettsia*) in cicadas, which may provide new information about the transmission strategies of symbionts in cicadas and other auchenorrhynchan insects. Manipulation of symbiotic organs or microbial symbiont(s) seems to be a novel strategy for the control of insect pests especially for plant sap-feeding insects [48–50]. Studies on the diversification and transovarial transmission of symbionts in cicadas provide not only novel insights into uncovering complicated host-symbiont associations but also helpful guidelines for the control of sap-feeding pests.

### The transmission dynamics of symbionts in cicadas

Among the sap-sucking hemipterans, specific symbionts are generally hosted within the bacteriomes and/or fat bodies [51–54]. Obligatory symbionts hosted in the specialized symbiotic organs tend to be conserved in the evolutionary process due to the intimate host-symbiont associations, such as strictly transovarial transmission of these symbionts [54]. However, such mutualistic symbionts may have been lost or replaced occasionally, which may affect the development and evolution of the symbiotic organs in the host insects [54].

It is generally thought that auchenorrhynchan ancestor has been colonized by *Sulcia* and a coresident betaproteobacterium, whereas one or both symbiont(s) was complemented or replaced by other symbiont(s) during the evolution process of some sap-feeding groups [12]. A previous study has shown that *Sulcia* and the coresident bacterial symbiont(s) have been completely lost and replaced by YLS in leafhoppers *L. auditura* and *Ti. angulata*, and the bacteriomes are even absent in these two leafhoppers [32]. These observations uncover intimate associations between the microbial symbionts and the evolution of the symbiotic organs. We revealed that the composition of symbionts in Cicadidae can be divided into 8 categories, among which two categories are novel (Fig. 2). Our results demonstrate the symbiotic systems in Cicadidae are diverse, and show complex distribution patterns of obligate symbionts in the bacteriomes and fat bodies (Figs. 2 and S3). We revealed that YLS is harbored in the fat bodies of adults of *K*. *caelatata* and *Ta.* sp., whereas in both the fat bodies and bacteriome sheath of adults of *G. tienta* and* H*. *maculaticollis* (Fig. S3). A previous study reported that YLS was only harbored in the bacteriome sheath of *Graptopsaltria nigrofuscata* adults [20], but it still needs to be confirmed. Our present study and other previous studies show that *Sulcia* is found in the bacteriomes of all investigated cicada species to date. However, we cannot rule out the possibility that both *Sulcia* and *Hodgkinia* have been completely replaced by YLS in some species of Cicadidae, in which the bacteriomes may become vestigial or even absent.

It has been reported that YLS only colonizing the fat bodies seems to be more common than colonizing both the fat bodies and bacteriome sheath in adults of *Hodgkinia*-free cicadas [20, 30, 31]. We revealed that *Sulcia*, *Hodgkinia*, YLS and a couple of facultative symbionts can be transovarially transmitted to mature oocytes, which form a “symbiont ball” in each egg. In regard to *Hodgkinia*-free cicadas, YLS is initially harbored together with *Sulcia* in the “symbiont ball” in eggs, and then segregated by the bacteriomes in young-instar nymphs, but finally migrates to the fat bodies of adults in the majority of *Hodgkinia*-free species, whereas YLS cells reside in both the bacteriome sheath and fat bodies of adults in a few other species (e.g., *H. maculaticollis* and *G. tienta*) (Fig. 6). These results suggest that the distribution of endosymbionts is closely related to the development of symbiotic organs, such as bacteriomes and fat bodies. A previous study hypothesized that fat bodies may not be the most suitable organs for YLS to colonize and, therefore, YLS may evolve to occupy the bacteriocytes of bacteriomes in related cicada species [30]. However, our results suggest that YLS may evolve to reside in the fat bodies rather than the bacteriomes in *Hodgkinia*-free species, which could provide new insights into the evolution of symbiotic organs.

## Conclusions

Collectively, our results revealed novel distribution of symbionts in bacteriomes and fat bodies during the development of cicadas. *Sulcia*, *Hodgkinia*, YLS and *Arsenophonus* can be transovarially transmitted to mature oocytes, which form a “symbiont ball” in each egg. The transmitted *Sulcia*/YLS or *Sulcia*/*Hodgkinia* cell number ratio varying significantly among species could be related to the distribution and/or lineage splitting of related symbiont(s). YLS is initially harbored together with *Sulcia* in the “symbiont ball” in eggs, and then segregated from other host tissues by the bacteriomes in young-instar nymphs, but YLS finally migrates to the fat bodies of adults in the majority of *Hodgkinia*-free species, whereas it resides in both the bacteriome sheath and fat bodies of adults in a few other cicada species. These results uncover the transmission dynamics of symbionts in cicadas and other related auchenorrhynchan insects. Our results provide novel insights into insect-microbe symbiosis and the evolution of symbiotic organs in sap-feeding insects.

## Materials and methods

### Sample collection and dissection

Cicada specimens were collected during the adult emergence period between 2016 and 2022, and detailed sample information has been listed in Table S5. Cicadas were identified based on morphological characters and DNA barcode (*COI*). Specimens were subsequently transferred to the laboratory for dissection. A detailed description of the dissection procedure is the same as previously described [25]. The dissected samples were used for DNA extraction and fluorescence and electron microscopy (Table S6).

### Microbial composition of bacteriomes and fat bodies analyzed by RFLP-based cloning and phylogenetic analysis of obligate symbionts

PCR amplification for the 16S rRNA/18S rRNA gene of microbial symbionts was performed as previously described [20, 25]. The PCR products were purified with a Universal DNA Purification Kit (Tiangen Inc.), cloned into pMD™ 19-T Vector (Takara Inc.) and subsequently transformed into *Escherichia coli* DH5α Competent Cells (Takara Inc.). For each biological replication, ~ 200 positive clones were selected randomly from a white-blue selection system containing ampicillin and X-gal, and then grown overnight in 900 μl liquid lysogeny broth (LB) medium at 37 °C. Extracted plasmid DNA was used as the template for subsequent PCR amplification with M13 vector primers to check positive clones. If the PCR product was the expected size of nearly 1.5 kb, it was digested with restriction endonucleases *Hha I* at 37 °C for 3 h. The restriction fragments were separated by 1% agarose gel electrophoresis and analyzed under UV light. Representative clones for each RFLP profile were randomly selected for sequencing at Sangon Biotech Co., Ltd (Shanghai, China). The 16S rRNA gene sequences of bacterial symbionts and 18S rRNA gene sequences of fungal symbionts were used as queries in BLAST searches through NCBI GenBank nucleotide database, Ribosomal Database Project (RDP), Silva Database and Greengenes Database.

The accession numbers of 16S rRNA/18S rRNA gene sequences of microbial symbionts downloaded from NCBI GenBank nucleotide database were listed in Table S7. Multiple alignments of the 16S rRNA/18S rRNA gene sequences of the symbiont genes were performed using the program Clustal X [55]. Subsequently, BioEdit software was used to remove the gap-containg sites and ambiguously aligned sites at the beginning and end of the aligned sequences [56]. The phylogenetic trees of the 16S rRNA gene sequences of *Sulcia* and *Hodgkinia,* and 18S rRNA gene sequences of the YLS were reconstructed using the PhyloSuite software [57].

### Diagnostic PCR analyses of dominant symbionts in different tissues

Diagnostic PCR amplification was performed to confirm the presence of dominant symbionts in the fat bodies and bacteriomes. PCR primers and cycle conditions used for the amplification of *Sulcia* and YLS were as previously described [12, 20]. The PCR primers used in this study are summarized in Table S8. The PCR products were determined by 1% agarose gel electrophoresis and then purified with a Universal DNA Purification Kit (BioTeke Inc.). Representative PCR products were sent for sequencing at Sangon Biotech Co., Ltd (Shanghai, China). The sequences obtained by diagnostic PCR amplification were used as queries in BLAST searches through NCBI GenBank nucleotide database, Ribosomal Database Project (RDP), Silva Database and Greengenes Database.

### Fluorescence in situ hybridization

The dissected bacteriomes, fat bodies and ovaries were fixed in 4% paraformaldehyde, dehydrated in a graded ethanol series, cleared in xylene, and finally embedded with melted paraffin. Paraffin blocks were sectioned to 4 μm, and thin sections were used for histological or fluorescence microscopy. Histological sections were stained with hematoxylin and eosin.

The fluorescence in situ hybridization (FISH) was conducted to reveal the distribution of symbionts in the fat bodies, bacteriomes and ovaries of different cicada species. The FISH assay was performed as previously described [25, 58, 59]. The probe and helper sequences used in this study were listed in Table S9. Briefly, each slide was carried out in a final volume of 25 μl hybridization buffer contained 2.5X SSC (1X SSC is 0.15 M NaCl plus 0.015 M sodium citrate), 12.5% dextran sulfate, 0.25% bovine serum albumin (BSA), and fluorescently labeled probes (200 nM). Hybridization was conducted in a 37 °C humidified chamber for ~ 10 h. In addition, negative control was done using no probe staining and only one symbiont-targeted probe to check the specificity of hybridization. Slides were observed and imaged under an Olympus FV 3000 IX inverted laser scanning confocal microscope (Olympus, Japan).

### Histological and ultrastructural microscopy

The bacteriomes, fat bodies, ovaries, eggs and first instar nymphs were dissected in 0.1 M phosphate-buffered saline (PBS, pH 7.2), and subsequently fixed in 2.5% glutaraldehyde in 0.1 M PBS (pH 7.2) at 4 °C overnight. After rinsing with PBS for five times, the samples were post-fixed with 1% osmium tetroxide (OsO_4_) in 0.1 M PBS for 1.5 h at 4 °C. After washing with PBS for six times, the samples were dehydrated in a graded ethanol series (30%, 50%, 70%, 80%, 90% for 10 min twice, 95% for 15 min twice, and 100% for 30 min twice). Then, the samples were infiltrated with a graded mixture of ethanol and LR White, subsequently with LR White for 24 h twice, and eventually embedded in pure LR White, polymerized at 60 °C for 48 h.

Semithin sections (1 μm) were stained with 1% methylene blue and photographed under a light microscope Leica DM6 B (Leica, Germany). Ultrathin sections (70 nm) were double stained with uranyl acetate and lead citrate, and finally examined under the FEI Tecnai G2 Spirit Bio TWIN (FEI, Czech Republic). In our present study, it is quite difficult to make precise estimates of total symbiont cell numbers transmitted to eggs. Statistical analysis was performed using the semithin sections of mature oocytes to count the transmitted obligate symbiont cell numbers under histological microscopy with a 100X lens, aiming to estimate the ratio of cell numbers of transmitted obligate symbionts in different cicadas.

### Supplementary Information

Supplementary Material 1.

## Data Availability

All sequences have been deposited in the NCBI nucleotide database under the accession numbers: ON891652–ON891657, ON907639–ON907641, OP851576–OP851583, OP851564–OP851575, OP852131–OP852136, PP854659–PP854662 and PP849678–PP849680.
