# Supplementary material for: Segregation of endosymbionts in complex symbiotic system of cicadas providing novel insights into microbial symbioses and evolutionary dynamics of symbiotic organs in sap-feeding insects
Source: Front Zool. 2024 Jun 11;21:15. doi: 10.1186/s12983-024-00536-0 (PMC11165832; doi:10.1186/s12983-024-00536-0)
Supplement: Supplementary file 1 — Supplementary Material 1. [file 12983_2024_536_MOESM1_ESM.docx]

**Supplemental materials**

**Table S1** Morphological features of bacteriomes in different cicadas

| Host cicada | Unit numbers of bacteriomes (Mean ± SD) | Biological replicates of individuals | Thickness of bacteriome sheath (Mean ± SD) | Biological replicates of bacteriome units |
| --- | --- | --- | --- | --- |
| *Eopycna coelestia* | 99 ± 9 | 10 | 19.57 ± 4.05 μm | 20 |
| *Tettigetta* sp. | 12 ± 2 | 10 | 13.27 ± 1.79 μm | 20 |
| *Karenia caelatata* | 276 ± 15 | 8 | 13.53 ± 2.95 μm | 20 |
| *Tanna* sp. | 116 ± 9 | 10 | 13.60 ± 2.95 μm | 20 |
| *Graptopsaltria tienta* | 367 ± 12 | 10 | 60.76 ± 6.42 μm | 20 |
| *Hyalessa maculaticollis* | 235 ± 13 | 7 | 39.23 ± 6.51 μm | 20 |

**Table S2** Prevalence of dominant symbionts in bacteriomes and fat bodies of different cicadas using diagnostic PCR amplifications

| **Symbiont** | Numbers of positive individuals/total numbers of individuals tested | | | | | | | | | | | |
| --- | --- | --- | --- | --- | --- | --- | --- | --- | --- | --- | --- | --- |
|  | *Karenia caelatata* | | *Tanna* sp. | | *Graptopsaltria tienta* | | *Hyalessa maculaticollis* | | *Eopycna coelestia* | | *Tettigetta* sp. | |
|  | Ba | Fb | Ba | Fb | Ba | Fb | Ba | Fb | Ba | Fb | Ba | Fb |
| *Sulcia* | 10/10 | / | 10/10 | / | 10/10 | / | 10/10 | / | 10/10 | / | 10/10 | / |
| *Hodgkinia* | / | / | / | / | / | / | / | / | 10/10 | / | 10/10 | / |
| YLS | / | 10/10 | / | 10/10 | 10/10 | 10/10 | 10/10 | 10/10 | / | / | / | / |
| *Arsenophonus* | / | / | / | / | / | / | / | / | / | 10/10 | / | / |
| *Rickettsia* | 10/10 | 10/10 | / | / | / | / | / | / | / | / | / | 10/10 |

Note: The “/” represents the absence of symbiont in these tissues. Ba, bacteriomes; Fb, fat bodies.

**Table S3** The RFLP analysis revealing the distribution of *Sulcia*, *Hodgkinia* and YLS in the bacteriomes and fat bodies of different cicadas

| Host cicada | Tissue(s) | Clone number | Closest match in Databases | Identity (%) |
| --- | --- | --- | --- | --- |
| *Karenia caelatata* | bacteriomes | Clone-KA2 | *Sulcia* | 99.93% |
| *Tanna* sp. | bacteriomes | Clone-TA3 | *Sulcia* | 99.40% |
| *Graptopsaltria tienta* | bacteriomes | Clone-GT5 | *Sulcia* | 99.93% |
| *Hyalessa maculaticollis* | bacteriomes | Clone-HY9 | *Sulcia* | 99.93% |
| *Eopycna coelestia* | bacteriomes | Clone-EC26 | *Sulcia* | 99.73% |
| *Tettigetta* sp. | bacteriomes | Clone-Tesp8 | *Sulcia* | 99.93% |
| *Karenia caelatata* | fat bodies | Clone-ka5 | YLS | 100% |
| *Tanna* sp. | fat bodies | Clone-ta8 | YLS | 99.92% |
| *Graptopsaltria tienta* | fat bodies, bacteriomes | Clone-gt9 | YLS | 99.67% |
| *Hyalessa maculaticollis* | fat bodies, bacteriomes | Clone-hy1 | YLS | 100% |
| *Eopycna coelestia* | bacteriomes | Clone-EC3 | *Hodgkinia* | 99.91% |
| *Eopycna coelestia* | bacteriomes | Clone-EC7 | *Hodgkinia* | 99.93% |
| *Eopycna coelestia* | bacteriomes | Clone-EC9 | *Hodgkinia* | 96.99% |
| *Eopycna coelestia* | bacteriomes | Clone-EC34 | *Hodgkinia* | 99.91% |
| *Eopycna coelestia* | bacteriomes | Clone-EC62 | *Hodgkinia* | 98.62% |
| *Eopycna coelestia* | bacteriomes | Clone-EC112 | *Hodgkinia* | 97.26% |
| *Eopycna coelestia* | bacteriomes | Clone-EC138 | *Hodgkinia* | 99.93% |
| *Eopycna coelestia* | bacteriomes | Clone-EC140 | *Hodgkinia* | 96.99% |
| *Tettigetta* sp. | bacteriomes | Clone-Tesp2 | *Hodgkinia* | 96.50% |
| *Tettigetta* sp. | bacteriomes | Clone-Tesp13 | *Hodgkinia* | 98.38% |
| *Tettigetta* sp. | bacteriomes | Clone-Tesp15 | *Hodgkinia* | 96.90% |
| *Tettigetta* sp. | bacteriomes | Clone-Tesp32 | *Hodgkinia* | 97.77% |
| *Tettigetta* sp. | bacteriomes | Clone-Tesp38 | *Hodgkinia* | 98.65% |
| *Tettigetta* sp. | bacteriomes | Clone-Tesp42 | *Hodgkinia* | 97.70% |
| *Tettigetta* sp. | bacteriomes | Clone-Tesp60 | *Hodgkinia* | 98.24% |
| *Tettigetta* sp. | bacteriomes | Clone-Tesp82 | *Hodgkinia* | 99.79% |
| *Tettigetta* sp. | bacteriomes | Clone-Tesp91 | *Hodgkinia* | 98.17% |
| *Tettigetta* sp. | bacteriomes | Clone-Tesp92 | *Hodgkinia* | 99.74% |
| *Tettigetta* sp. | bacteriomes | Clone-Tesp115 | *Hodgkinia* | 98.38% |
| *Tettigetta* sp. | bacteriomes | Clone-Tesp123 | *Hodgkinia* | 99.79% |

**Table S4** PCR amplification revealing the distribution of facultative symbionts in the bacteriomes and fat bodies of different cicadas

| Host cicada | Tissue(s) | Closest match in Database | Identity (%) |
| --- | --- | --- | --- |
| *Eopycna coelestia* | fat bodies | *Arsenophonus* | 97.67% |
| *Karenia caelatata* | fat bodies, bacteriomes | *Rickettsia* | 99.68% |
| *Tettigetta* sp. | fat bodies | *Rickettsia* | 99.82% |

**Table S5** The adult cicada samples used in this study

| Species | Stage | Locality | Collection date |
| --- | --- | --- | --- |
| *Karenia caelatata* | adults | Ningshan County, Shaanxi Province | middle August of 2016-2021 |
| *Hyalessa maculaticollis* | eggs, nymphs, aduts | Ningshan County, Shaanxi Province | late July of 2020-2021 |
| *Tanna* sp. | adults | Ningshan County, Shaanxi Province | late July of 2016-2021 |
| *Graptopsaltria tienta* | adults | Emeishan Mountains, Sichuan Province | middle August of 2019-2021 |
| *Eopycna coelestia* | adults | Tongjiang County, Sichuan Province | middle April of 2020-2021 |
| *Tettigetta* sp. | adults | Fufeng County, Shaanxi Province | late June of 2020-2021 |
| *Cryptotympana atrata* | eggs, nymphs, aduts | Ningshan County, Shaanxi Province | Late July of 2021-2022 |
| *Eopycna repanda* | eggs, nymphs, aduts | Fengxian County, Shaanxi Province | Middle October of 2016-2021 |

**Table S6** The details of samples used for molecular analysis and fluorescence and histological and electron microscopy

| Species | RFLP analysis | PCR amplification | Fluorescence microscopy | Histological and electron microscopy |
| --- | --- | --- | --- | --- |
| *Karenia caelatata* | 3 females | 5 males, 5 females | 5 males, 20 females | 5 males, 20 females |
| *Hyalessa maculaticollis* | 3 females | 5 males, 5 females | 3 males, 23 females | 18 eggs; 20 nymphs; 3 males, 23 females |
| *Tanna* sp. | 3 females | 5 males, 5 females | 5 males, 18 females | 5 males, 18 females |
| *Graptopsaltria tienta* | 3 females | 5 males, 5 females | 3 males, 16 females | 3 males, 16 females |
| *Eopycna coelestia* | 3 females | 5 males, 5 females | 3 males, 12 females | 3 males, 12 females |
| *Tettigetta* sp. | 3 females | 5 males, 5 females | 3 males, 18 females | 3 males, 18 females |
| *Cryptotympana atrata* | \ | \ | \ | 16 eggs; 20 nymphs; 3 females |
| *Eopycna repanda* | \ | \ | \ | 20 eggs; 20 nymphs; 3 females |

**Table S7** The list of sequences downloaded from the NCBI GenBank nucleotide database

| **Symbiont** | **Host species** | **Accession number** |
| --- | --- | --- |
| *Sulcia* | *Ambragaeana sticta* | MT533402 |
| *Sulcia* | *Auritibicen bihamatus* | LC370637 |
| *Sulcia* | *Auritibicen jai* | MT533427 |
| *Sulcia* | *Auritibicen japonicus* | LC370637 |
| *Sulcia* | *Cryptotympana atrata* | MT533420 |
| *Sulcia* | *Cryptotympana facialis* | LC370653 |
| *Sulcia* | *Cryptotympana holsti* | MT533423 |
| *Sulcia* | *Dundubia hainanensis* | MT533373 |
| *Sulcia* | *Eopycna repanda* | MK770419 |
| *Sulcia* | *Euterpnosia chibensis* | LC370698 |
| *Sulcia* | *Gaeana maculata* | MT533404 |
| *Sulcia* | *Graptopsaltria bimaculata* | LC370690 |
| *Sulcia* | *Graptopsaltria nigrofuscata* | LC370560 |
| *Sulcia* | *Huechys thoracica* | MT533445 |
| *Sulcia* | *Katoa paucispina* | MT533439 |
| *Sulcia* | *Kosemia yezoensis* | LC370763 |
| *Sulcia* | *Macrosemia umbrata* | MT533363 |
| *Sulcia* | *Magicicada septendecim* | DQ066625 |
| *Sulcia* | *Magicicada tredecim* | CP010828 |
| *Sulcia* | *Meimuna iwasakii* | LC370740 |
| *Sulcia* | *Meimuna kuroiwae* | LC370736 |
| *Sulcia* | *Meimuna mongolica* | MT533379 |
| *Sulcia* | *Meimuna opalifera* | LC370726 |
| *Sulcia* | *Meimuna oshimensis* | LC370731 |
| *Sulcia* | *Mogannia conica* | MT533433 |
| *Sulcia* | *Mogannia cyanea* | MT533438 |
| *Sulcia* | *Mogannia indigotea* | MT533437 |
| *Sulcia* | *Mogannia minuta* | LC370590 |
| *Sulcia* | *Muda kuroiwae* | LC370764 |
| *Sulcia* | *Platypleura kaempferi* | MT533431 |
| *Sulcia* | *Pomponia linearis* | MT533387 |
| *Sulcia* | *Purana gigas* | MT533395 |
| *Sulcia* | *Subpsaltria yangi* | MT533428 |
| *Sulcia* | *Tanna japonensis* | LC370573 |
| *Sulcia* | *Terpnosia nigricosta* | LC370697 |
| *Sulcia* | *Terpnosia vacua* | LC370693 |
| *Sulcia* | *Tettigades undata* | CP007234 |
| YLS | *Ambragaeana sticta* | MT537696 |
| YLS | *Angamiana yunnanensis* | MT537677 |
| YLS | *Cryptotympana atrata* | MT537666 |
| YLS | *Cryptotympana facialis* | LC370815 |
| YLS | *Cryptotympana holsti* | LC370699 |
| YLS | *Euterpnosia chibensis* | LC370888 |
| YLS | *Euterpnosia okinawana* | LC370900 |
| YLS | *Gaeana maculata* | MT537688 |
| YLS | *Graptopsaltria bimaculata* | LC370865 |
| YLS | *Graptopsaltria nigrofuscata* | LC370844 |
| YLS | *Huechys thoracica* | MT537692 |
| YLS | *Meimuna iwasakii* | LC370967 |
| YLS | *Meimuna kuroiwae* | LC370966 |
| YLS | *Meimuna mongolica* | MT537654 |
| YLS | *Meimuna opalifera* | MT537653 |
| YLS | *Meimuna oshimensis* | LC370953 |
| YLS | *Mogannia conica* | MT537690 |
| YLS | *Mogannia indigotea* | MT537685 |
| YLS | *Mogannia minuta* | LC370999 |
| YLS | *Pomponia linearis* | MT537636 |
| YLS | *Purana gigas* | MT537704 |
| YLS | *Tanna japonensis* | LC370916 |
| YLS | *Terpnosia nigricosta* | LC370879 |
| YLS | *Terpnosia vacua* | LC370866 |
| YLS | *Subpsaltria yangi* | MT537670 |
| *Hodgkinia* | *Auritibicen bihamatus* | LC370505–LC370516 |
| *Hodgkinia* | *Auritibicen japonicus* | LC370531, LC370533, LC370538, LC370540  LC370541, LC370543, LC370545, LC370547 |
| *Hodgkinia* | *Eopycna repanda* | MK926459–MK926463 |
| *Hodgkinia* | *Kosemia yezoensis* | LC370599–LC370601, LC370604–LC370606  LC370611–LC370612 |
| *Hodgkinia* | *Magicicada septendecim* | KR607377 |
| *Hodgkinia* | *Magicicada tredecim* | KR607416 |
| *Hodgkinia* | *Platypleura kaempferi* | LC370452–LC370453, LC370456–LC370458  LC370460–LC370461, LC370463, LC370465  LC370467, LC370470–LC370472, LC370768  LC370770–LC370771 |
| *Hodgkinia* | *Tettigades undata* | CP007232–CP007233 |

**Table S8** The primers for amplification of dominant symbionts in this study

| **Target** | **Primer name** | **Primer sequence (5**'–**3**'**)** | **References** |
| --- | --- | --- | --- |
| *Sulcia*  (16S rRNA) | 10_CFB_FF | AGAGTTTGATCATGGCTCAGGATG | 12 |
|  | 1515_R | GTACGGCTACCTTGTTACGACTTAG |  |
| YLS  (18S rRNA) | Fng18S_82F | GAAACTGCGAATGGCT | 20 |
|  | NS4 | CTTCCGTCAATTCCTTTAAG |  |
| YLS  (18S rRNA) | Fng18S_82F | GAAACTGCGAATGGCT | 20 |
|  | Fng18S_1067R | TMTCGTAAGGTGCCGA |  |
| YLS  (18S rRNA) | Hyp325F | GTTTGGGTAGTGGCCAAAC | 20 |
|  | Hyp760R | CCTGCCTGGAGCACTCT |  |
| YLS | CFS2-CS | TTCAACGAGGAAYCCCTA | 20 |
| (18S rRNA) | LSU_CS-1R | GCTTCACTCGCCGTTAC |  |
| YLS | ITS_CS-1F | CATCGAATCTTTGAACGC | 20 |
| (28S rRNA) | NLB1_CS | CTTTACCTCATAAAACTGAGNTCG |  |
| YLS | RPB1-OhpF1 | GTCTWCCACCCGGGCTT | 20 |
| (RPB1) | RPB1-OhpR1 | GGCRATGTCGTTGTCCAT |  |
| YLS | RPB2-OphF1 | YAAGAAGCGYCTCGACCT | 20 |
| (RPB2) | RPB2-OphR2 | GGCAGACTGGTACGTGTT |  |
| YLS | Ef1α-OphF1 | CCGGCCACCGTGACTTCAT | 20 |
| (Ef1α) | Ef1α-Oph2218R | ATGACACCGACGGCRMCGGTYTG |  |
| *Arsenophonus* | Ars-F | CAATGGGCGAAAGCCTGATG | 25 |
| (16S rRNA) | Ars-R | ACCCCAGTCATGAACCACAAA |  |
| *Rickettsia* | Rick-F | GTGGGAATCTGCCCATCAGT | 25 |
| (16S rRNA) | Rick-R | GCAGTGTGTACAAGRCCCGA |  |

**Table S9** Probes used for fluorescence in situ hybridization of *Sulcia*, YLS, *Hodgkinia,* *Arsenophonus* and *Rickettsia* of cicadas

| Probe name | Fluorophore | Primer sequence (5'–3') | References |
| --- | --- | --- | --- |
| *Sulcia* | CY3 | CCACACATTCCAGTTACTCC | 24 |
| *Sulcia*-Lhelper | unlabelled | CCTCACTCTAGTTTATCAGTATCAATAGCACTT |  |
| *Sulcia*-Rhelper | unlabelled | GTTCTGTGTGATCTCTATGCATTTCACCGCT |  |
| YLS | CY5 | CCTGCCTGGAGCACTCT | 20 |
| YLS-Lhelper | unlabelled | CTAATGTATTCGAGCAT | This study |
| YLS-Rhelper | unlabelled | TTTTTCAAAGTAAAAGTCCCGT |  |
| *Hodgkinia-EC* | CY5 | CCAGTGTGGCTGTCCGT (for *Eopycna coelestia*) | This study |
| *Hodgkinia-TS* | CY5 | CCAATGTGGCTGGTCGT (for *Tettigetta* sp.) |  |
| *Hodgkinia*-Lhelper | unlabelled | CTCCCAGACCAGCTATAGATCGTTGCC |  |
| *Hodgkinia*-Rhelper | unlabelled | CCGTAGAAGTTTGGGCCGTGTCTCAGT |  |
| *Rickettsia* | Alexa488 | TCCACGTCACCGTCTTGC | This study |
| *Arsenophonus* | Alexa488 | TCTAGATTACAACCTCCAAA | This study |
| *Ars*-Lhelper | unlabelled | GACATCGTTTACAGCCTGG |  |
| *Ars*-Rhelper | unlabelled | CGTTAGCTTCGGAGGCCAC |  |

**
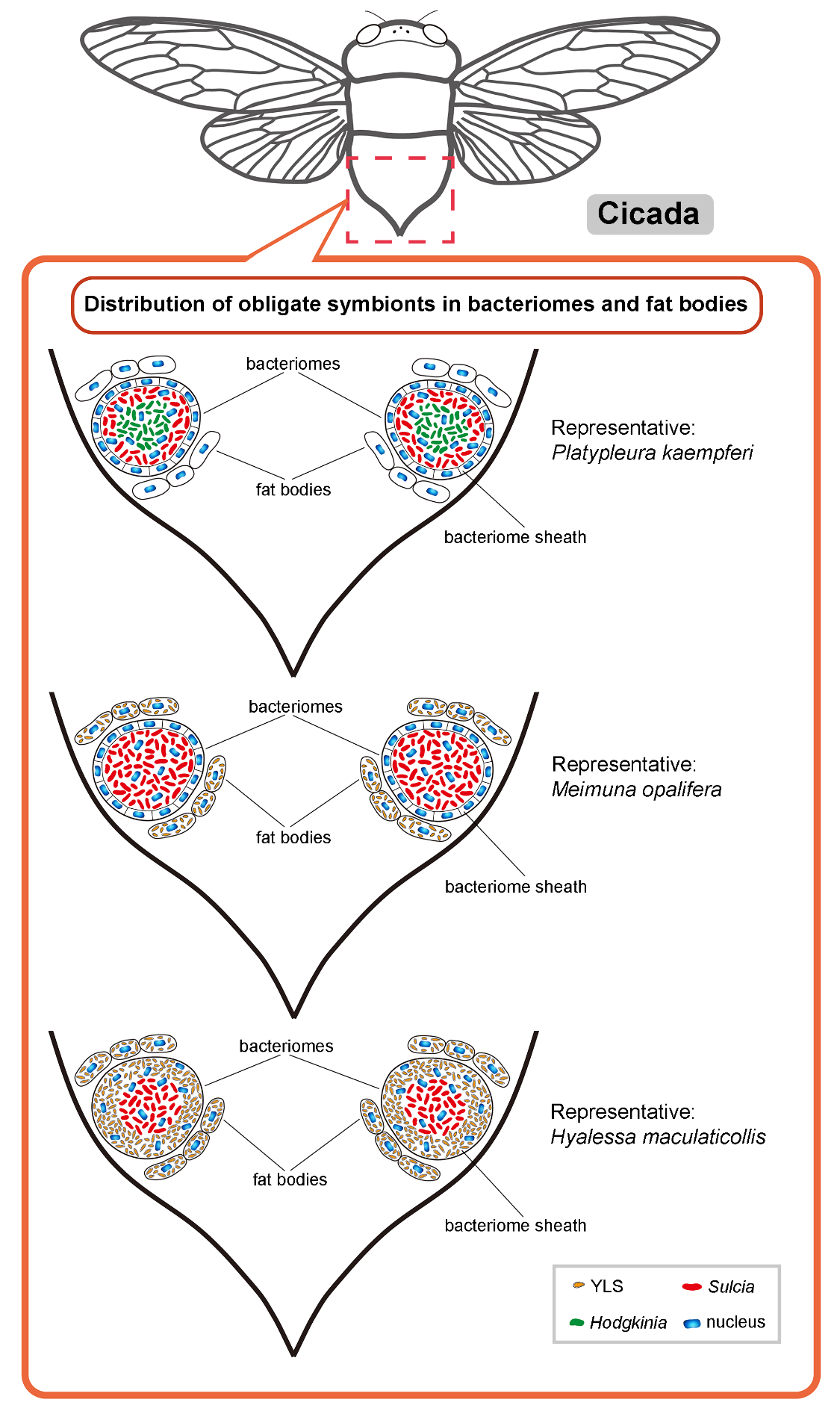
**

**Fig S1** Schematic representation showing the distribution of *Sulcia*, *Hodgkinia* and YLS in the bacteriomes and fat bodies of cicadas.


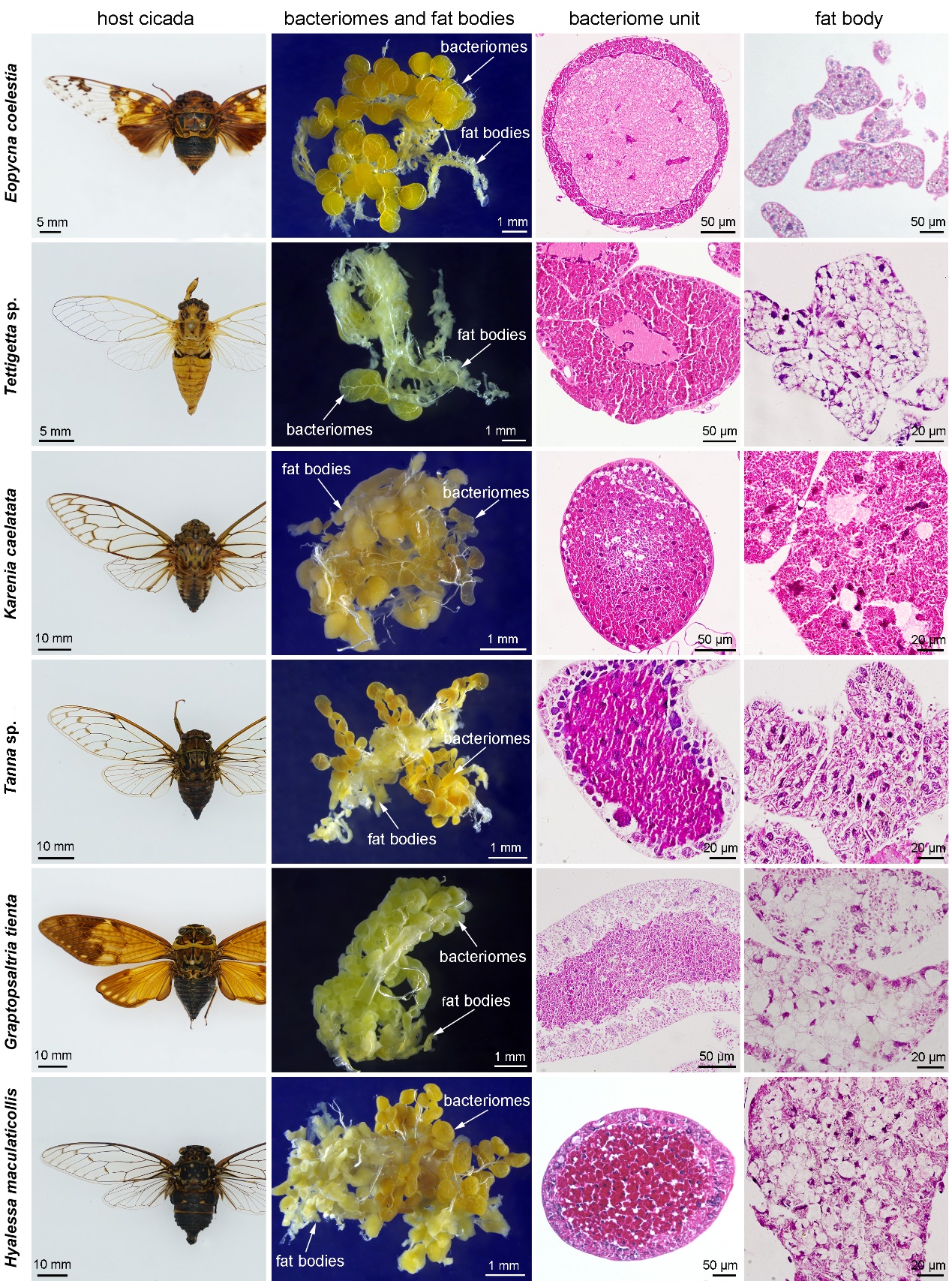


**Fig S2** General features of bacteriome units and fat bodies in adults of six different cicadas.


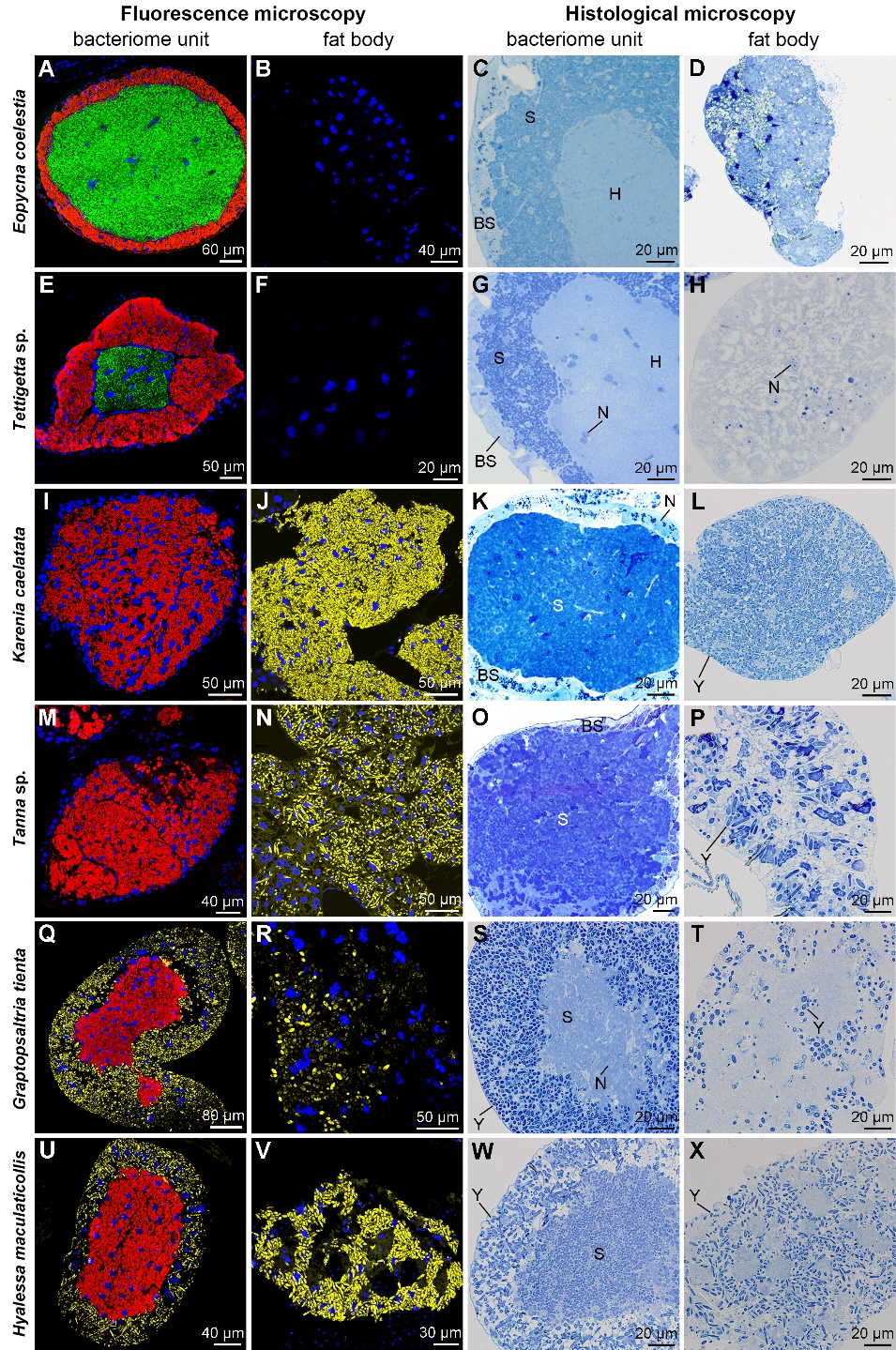


**Fig S3.** Distribution of obligate symbionts in the bacteriomes and fat bodies of six cicada species. (A to D) *Sulcia* and *Hodgkinia* in the bacteriomes of *Eopycna coelestia*. (E to H) *Sulcia* and *Hodgkinia* in bacteriomes of *Tettigetta* sp.. (I to L) *Sulcia* in the bacteriomes and YLS in the fat bodies of *Karenia caelatata*. (M to P) *Sulcia* in the bacteriomes and YLS in the fat bodies of *Tanna* sp.. (Q to T) *Sulcia* in the bacteriomes, while YLS in the fat bodies and bacteriome sheath of *Graptopsaltria tienta*. (U to X) *Sulcia* in the bacteriomes, while YLS in the fat bodies and bacteriome sheath of *Hyalessa maculaticollis*. For fluorescence microscopy, blue, yellow, red and green represent nucleus, YLS, *Sulcia* and *Hodgkinia*, respectively. Abbreviations: BS, bacteriome sheath; H, *Hodgkinia*; N, nucleus; S, *Sulcia*; Y, yeast-like fungal symbiont.


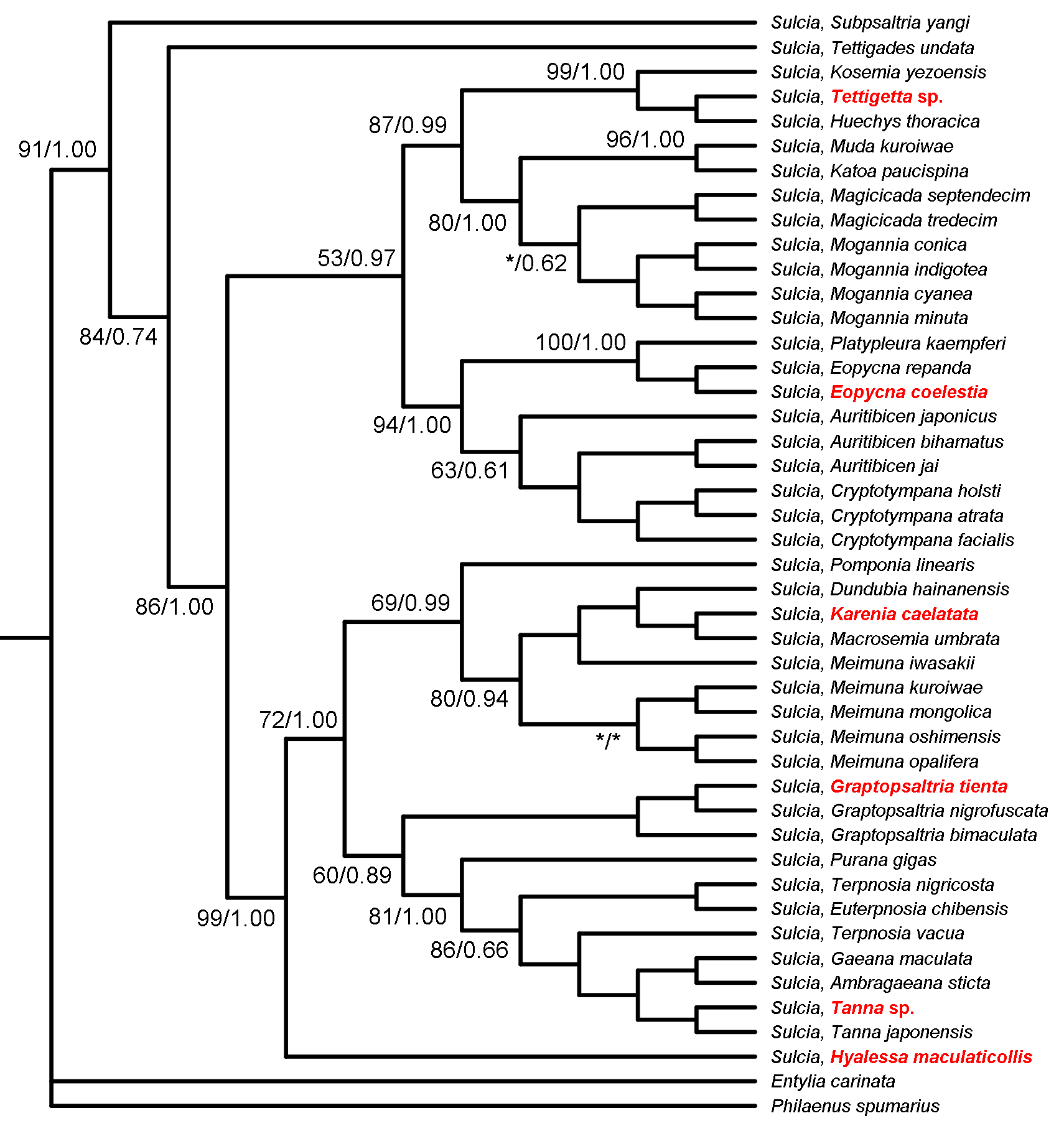


**Fig S4** Phylogenetic relationship inferred from bacterial 16S rRNA gene sequences of *Sulcia* symbionts of cicadas. Asterisks representing support values less than 50% and bootstrap support values more than 50% are shown on each node in the order of the maximum-likelihood/Bayesian inference. Bootstrap support values and posterior probabilities of the maximum-likelihood/Bayesian inference are shown near branches. Red font represents the cicada species investigated in this study.


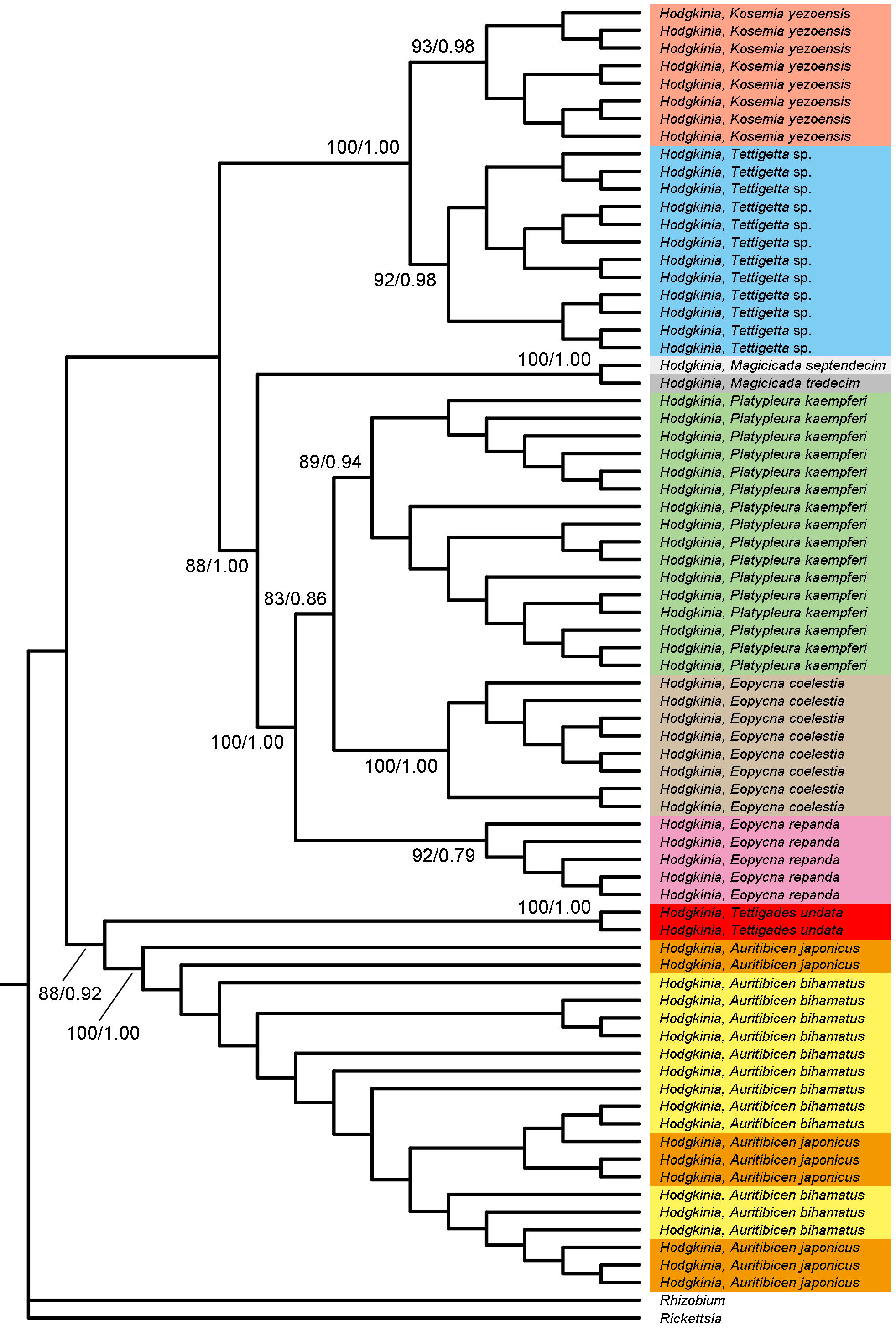


**Fig S5** Phylogenetic relationship inferred from bacterial 16S rRNA gene sequences of *Hodgkinia* symbionts of cicadas. Asterisks representing support values less than 50% and bootstrap support values more than 50% are shown on each node in the order of the maximum-likelihood/Bayesian inference. Bootstrap support values and posterior probabilities of the maximum-likelihood/Bayesian inference are shown near branches.


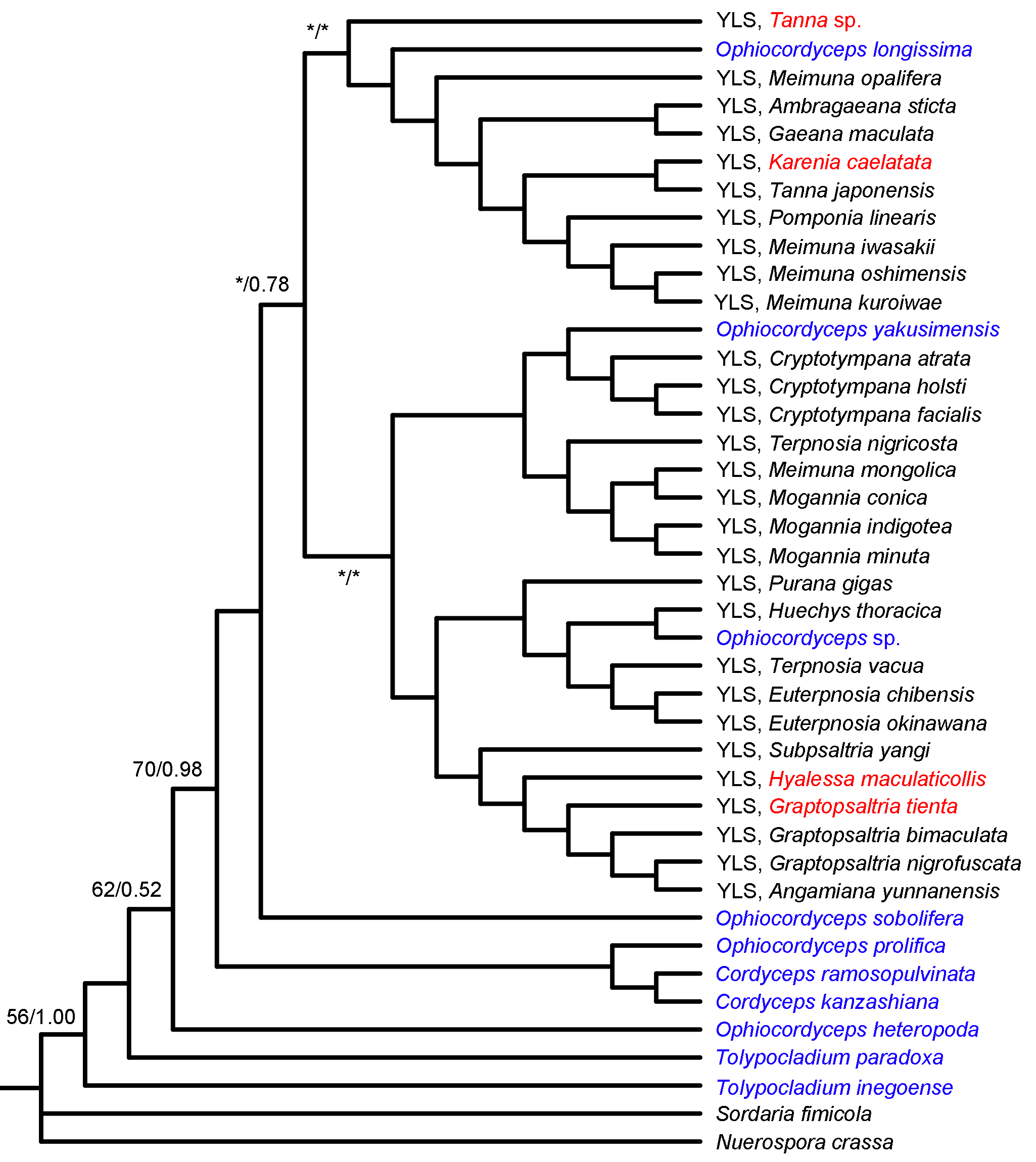


**Fig S6** Phylogenetic relationship inferred from bacterial 18S rRNA gene sequences of YLS symbionts of cicadas. Asterisks representing support values less than 50% and bootstrap support values more than 50% are shown on each node in the order of the maximum-likelihood/Bayesian inference. Bootstrap support values and posterior probabilities of the maximum-likelihood/Bayesian inference are shown near branches. Red font represents the cicada species used in this study. Blue font represents the parasite fungi of cicadas.


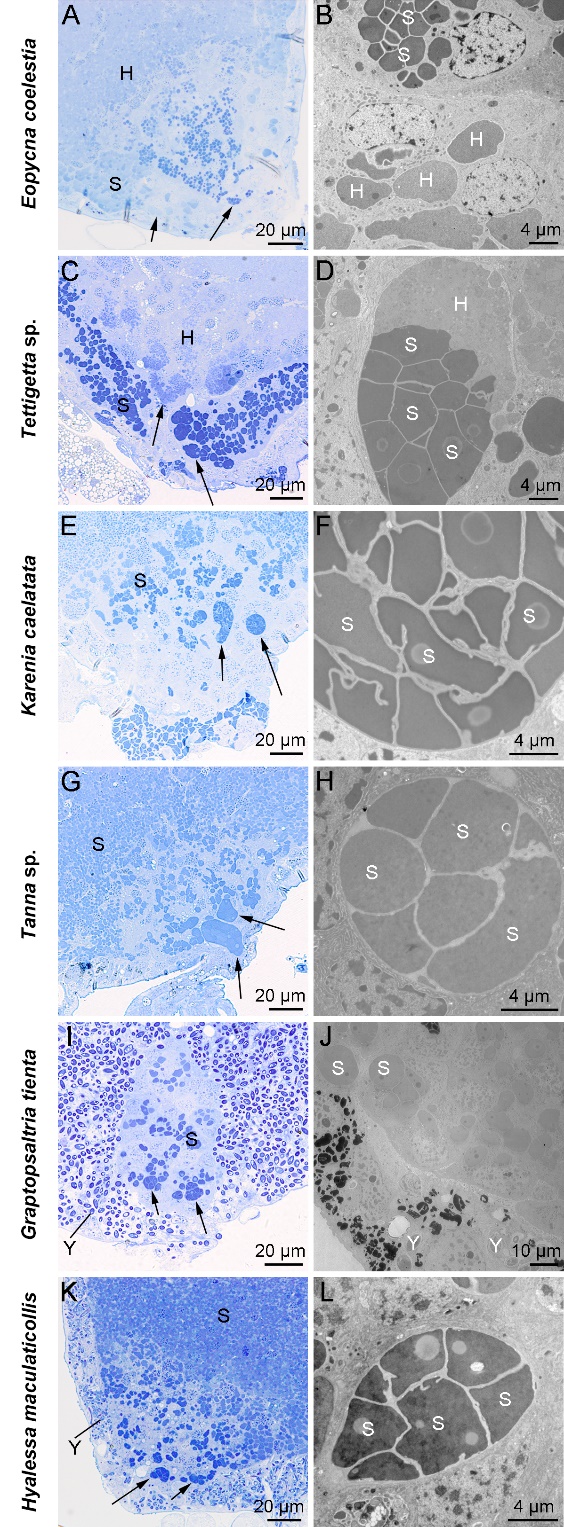


**Fig S7** The migration of symbiont cells from bacteriome unit to the hemolymph in mature females of different cicada species. (A to B) *Eopycna coelestia*. (C to D) *Tettigetta* sp.. (E to F) *Karenia caelatata*. (G to H) *Tanna* sp.. (I to J) *Graptopsaltria tienta*. (K to L) *Hyalessa maculaticollis*. Abbreviations: H, *Hodgkinia*; S, *Sulcia*; Y, yeast-like fungal symbiont. Black arrows indicate *Sulcia* or *Hodgkinia* cells in the bacteriomes gathered tightly before being released to the hemolymph.


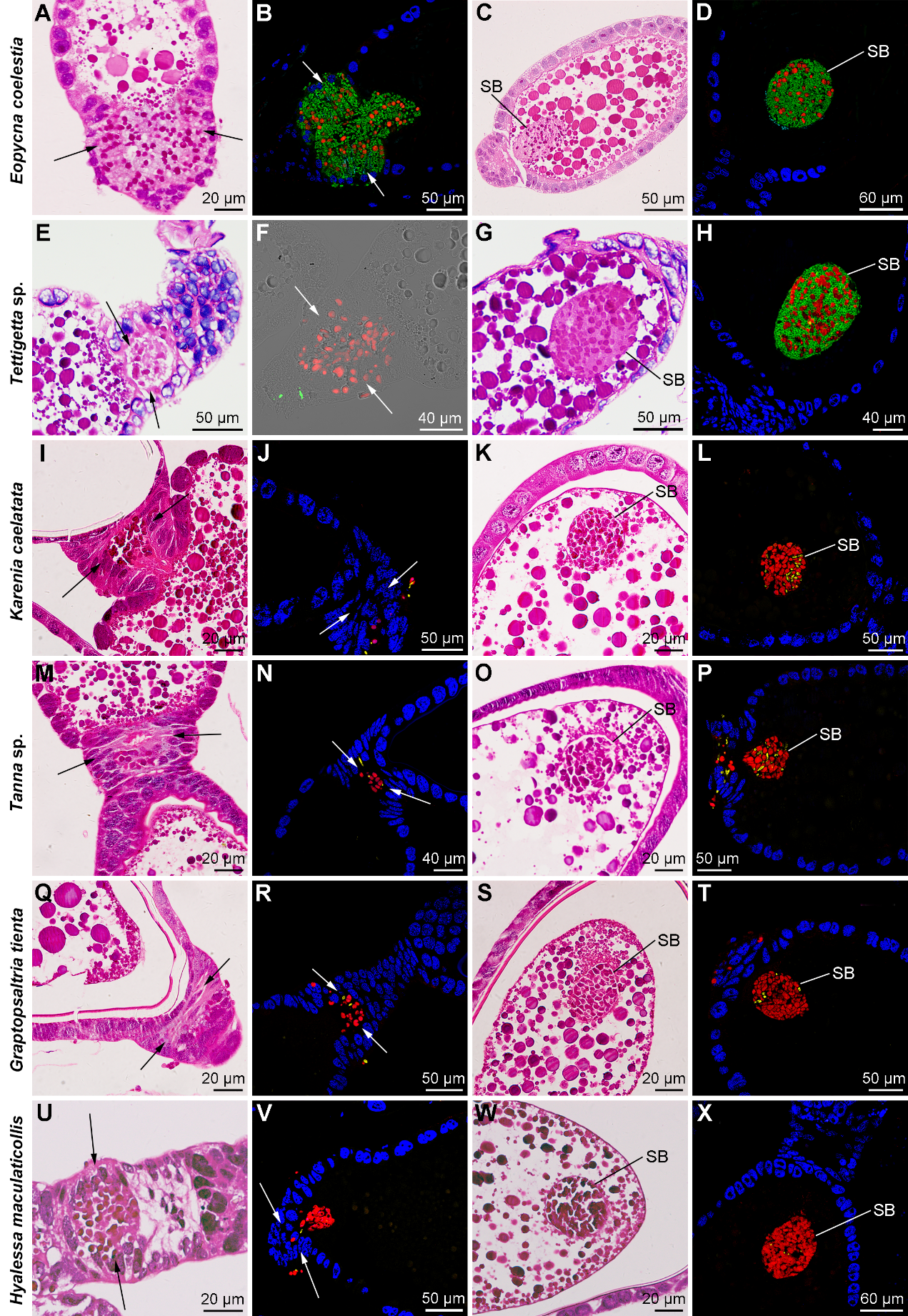


**Fig S8** Histological and fluorescent microscopy showing the transmission of symbionts from hemolymph to mature oocytes in different cicada species. (A to D) For *Eopycna coelestia*, *Arsenophonus* together with *Sulcia* and *Hodgkinia* were transported to the posterior pole of the mature oocytes, migrating through the cytoplasma of epithelial plug cells into the perivitelline space and finally formed a characteristic “symbiont ball” in each egg. (E to H) For *Tettigetta* sp., *Sulcia* and *Hodgkinia* were transported to the posterior pole of the mature oocytes, migrating through the cytoplasma of epithelial plug cells into the perivitelline space and finally formed a characteristic “symbiont ball” in each egg. (I to L) For *Karenia caelatata*, *Sulcia* and YLS were transported to the posterior pole of the mature oocytes, migrating through the cytoplasma of epithelial plug cells into the perivitelline space and finally formed a characteristic “symbiont ball” in each egg. (M to P) For *Tanna* sp., *Sulcia* and YLS were transported to the posterior pole of the mature oocytes, migrating through the cytoplasma of epithelial plug cells into the perivitelline space and finally formed a characteristic “symbiont ball” in each egg. (Q to T) For *Graptopsaltria tienta*, *Sulcia* and YLS were transported to the posterior pole of the mature oocytes, migrating through the cytoplasma of epithelial plug cells into the perivitelline space and finally formed a characteristic “symbiont ball” in each egg. (U to X) For *Hyalessa maculaticollis*, *Sulcia* and YLS were transported to the posterior pole of the mature oocytes, migrating through the cytoplasma of epithelial plug cells into the perivitelline space and finally formed a characteristic “symbiont ball” in each egg. For fluorescence microscopy, blue, cyan, yellow, red and green represent host nucleus, *Arsenophonus*, YLS, *Sulcia* and *Hodgkinia*, respectively. Black arrows representing the emigration of the symbionts from hemolymph to perivitelline space. Abbreviation: SB, symbiont ball.


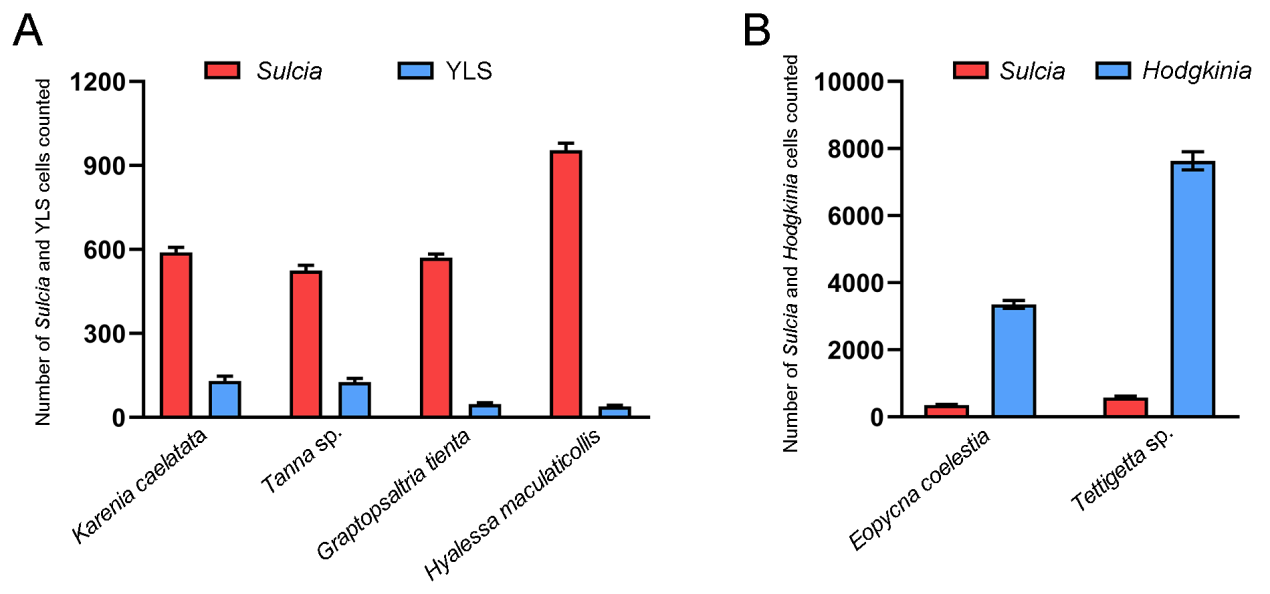


**Fig S9** Number of transmitted *Sulcia*, *Hodgkinia* and YLS cells in different cicada species. (A) Number of transmitted *Sulcia* and YLS cells counted in four *Hodgkinia*-free cicadas. (B) Number of transmitted *Sulcia* and *Hodgkinia* cells counted in *Tettigetta* sp. and *Eopycna coelestia*. Each sample contained eight individuals replicates.
